# Panama: An Open-Source Educational App for Ion Channel Biophysics Simulation

**DOI:** 10.3389/fninf.2022.813940

**Published:** 2022-03-09

**Authors:** Binita Rajbanshi, Anuj Guruacharya

**Affiliations:** ^1^Department of Epileptology, University Hospital Bonn, Bonn, Germany; ^2^Department of Biology, University of Oklahoma, Norman, OK, United States

**Keywords:** Hodgkin–Huxley simulation, web app for neuroscience, educational purposes, ion channels, biophysics

## Abstract

This article describes an open-source educational software, called Panama, developed using R, that simulates the biophysics of voltage-gated ion channels. It is made publicly available as an R package called Panama and as a web app at http://www.neuronsimulator.com. A need for such a tool was observed after surveying available software packages. Available packages are either not robust enough to simulate multiple ion channels, too complicated, usable only as desktop software, not optimized for mobile devices, not interactive, lack intuitive graphical controls, or not appropriate for educational purposes. This app simulates the physiology of voltage-gated sodium, potassium, and chlorine channels; A channel; M channel; AHP channel; calcium-activated potassium channel; transient-calcium channel; and leak-calcium channel, under current-clamp or voltage-clamp conditions. As the input values on the app are changed, the output can be instantaneously visualized on the web browser and downloaded as a data table to be further analyzed in a spreadsheet program. This app is a first-of-its-kind, mobile-friendly, and touchscreen-friendly online tool that can be used as an installable R package. It has intuitive touch-optimized controls, instantaneous graphical output, and yet is pedagogically robust for educational purposes.

## Introduction

The Hodgkin–Huxley (Hodgkin et al., 1952) model is one of the fundamental neuronal models. Its mathematical form is a set of differential equations that are used before moving on to more complex models. Computational simulations using this model strengthen the concepts of action potentials and ion channels.

Existing simulation programs, such as NEURON (Hines and Carnevale, 1997) and GENESIS (Bower et al., 2003), serve as powerful tools for simulating the response of whole-cell or single-channel parameters to electrical or pharmacological stimuli. Although such software tools are free and could be used for educational or research purposes, they require substantial training and may not be suitable for casual use by students with less computational knowledge. Some effort has been directed toward making educational packages that demonstrate ion channel biophysics that is freely available. These are good tools to know about action potentials, ion channel currents, and voltages. However, each of these tools has its own disadvantages. Some of them have been highlighted below.

HHsim^1^ (Touretzky et al., 2003) requires the software to be downloaded and a matching version of MATLAB installed on the desktop computer. Neurophysiology Virtual Lab^2^ (Sridharan et al., 2016) requires a signup procedure and is not mobile-friendly. NeuroLab^3^ (Schettino, 2014) requires a special software environment called Netlogo. Others, such as Phet,^4^ are cartoon reconstructions of ion channel physiology with restricted features. Nerve^5^ is not touch- or mobile-optimized whereas other programs (Molitor et al., 2006) are MATLAB packages. Any software package that is dependent on MATLAB is not ideal for wide distribution because of the overwhelming cost of MATLAB and the requirement to preinstall MATLAB. Also, any software package dependent on Java in the browser is not ideal because of the unavailability of built-in Java support in some modern web browsers.

Thus, a need was felt to make a tool that had the following characteristics: (1) mobile-friendly, (2) touchscreen-friendly, (3) pedagogically adequate for neurophysiological education, (4) completely online, (5) not reliant on MATLAB or Java software, (6) built with open-sourced code, and (7) usable by students that want an intuitive way to change ion channel parameters and download the data. To date, no electrophysiology simulation tool exists that satisfied all these criteria.

In this article, a new web app for simulating the biophysics of voltage-gated ion channels is described. It has been made publicly available at http://www.neuronsimulator.com and as a downloadable R package called Panama through GitHub. Its associated scripts are available at https://github.com/anuj2054/panama. R software is available at https://www.r-project.org/. Shiny Server is available at https://www.rstudio.com/products/shiny/. Lattice software is available at https://cran.r-project.org/web/packages/lattice/index.html. The design of the Panama software overcomes the limitations of previous simulators and satisfies all the criteria listed previously. R (R Core Team, 2014), Shiny package (Chang et al., 2017), and Lattice package (Sarkar, 2008) were used to code the software. It has multiple input controls for both voltage-clamp and current-clamp conditions. It outputs the voltage, current, and conductance values as graphs for each ion channel.

## Methods

### Numerical Design of the Simulator

The 11 channels simulated in this app were voltage-gated sodium, potassium, and chloride channels; calcium-activated potassium channels (*KCa*); T-type calcium channels (*CaT*); L-type calcium channels (*CaL*), leak sodium (*NaLeak*), and leak potassium (*KLeak*) channels; A current channels; M current channels; and AHP current channels. Each channel was represented by its maximal conductance or permeability (*g*_*n*_ or *p*_*n*_ where n is the specific ion channel), its ionic current (*I*_*n*_), its reversal potential (*E*_*n*_), and its associated gating parameters. Total ionic current (*I*_*net*_) was modeled as the sum of all those individual Hodgkin–Huxley style ionic currents: *i*_*Na*_, *i*_*K*_, *i*_*Cl*_, *i*_*NaLeak*_, *i_*KLeak*_,i_*A*_*, *i*_*M*_, *i*_*KCa*_, *i*_*AHP*_, *i*_*KCa*_, *i*_*M*_, *i*_*CaT*_, and *i*_*CaL*_. The models for these channels were modified from those used in the EOTN software (Campbell, 1996).

Voltage or current across the membrane was held constant depending on the clamping conditions. For the current-clamp case, *I*_*net*_ was held for the clamp duration at the applied current provided by the user; *V*_*net*_ was determined from Kirchhoff’s current law by solving a differential equation given in Equation 1. The membrane capacitance per area represented by *C* in Equation 1 is input by the user and set to a default value of 0.01 nFarads.


(1) $$\frac{dV_{net}}{dt}=\frac{1}{C}(I_{net}-i_{Na_{leak}}-i_{K_{leak}}-I_{Na}-i_{K}-i_{Cl}-i_{CaL}-i_{KCa}-i{\_}_{CaT}-i_{A}-i_{AHP}-i_{M})$$

For the voltage-clamp case, *V*_*net*_ was held for the clamp duration (set to a default of 50 ms) at the applied voltage provided by the user; *I*_*net*_ was determined from Kirchhoff’s current law as shown in Equation 2.


(2) $$I_{net}=(-i_{K_{leak}}-i_{K_{leak}}-I_{Na}-i_{K}-i_{Cl}-i_{CaL}-i_{KCa}-i{\_}_{CaT}-i_{A}-i_{AHP}-i_{M})$$

The default simulation time was set to 70 ms, with 10 ms being the preclamp and 10 ms being the postclamp duration. This helped to avoid data overflow issues. However, the clamp duration, preclamp duration, and postclamp duration can be changed to increase the total simulation time to 1,000 ms.

In all models, voltage was measured in *mVolt*, current in *nAmp*, time in *msec*, conductance in *μSiemens*, and capacitance in *nFarads*. We derived the model parameters for sodium channels from data of other groups (Huguenard et al., 1988). The potassium-channel model used was of a general delayed rectifier. T-type calcium channel (*i*_*CaT*_) was modeled using the constant field equation. L-type calcium channel (*i*_*CaL*_) was also modeled using the constant field equation as in *i*_*CaT*_, except that it was considered to not inactivate. *i*_*CaL*_ was based upon the data of another team (Kay and Wong, 1987) from isolated hippocampal pyramidal cells. Calcium-activated potassium channel (*i*_*KCa*_) was modeled according to the procedure used by other groups (Yamada, 1989). We also used the same group (Yamada, 1989) to model the AHP current according to the model in bullfrog sympathetic cells. The A current was modeled to inactivate with two-time constants. The first component consisting of *m1*_*A*_ and *h1*_*A*_ contributes 60% to the total value of the gating variables. The second component consisting of *m2*_*A*_ and *h2*_*A*_ contributes 40% to the total value of the gating variables. We adapted the M current model from other groups (Adams et al., 1982).

### Software Design of the Simulator

The web app was created using the R-programming language. After an initial survey of different languages and packages available in each language, the R language was chosen for its availability of Shiny and Lattice packages which are both excellent packages for web development and graphics development.

Euler’s method was the mathematical algorithm used to solve the differential equations. The differential equations were coded into the script using only R, without using any external differential equation solver packages, such as deSolve.

The Shiny package was used to serve the webpages. The Twitter bootstrap toolkit was used as the theme for user interface controls. Sliders from the bootstrap UI toolkit were used to make the input controls touch-friendly, so that users do not have to type the values in a textbox.

**FIGURE 1:**
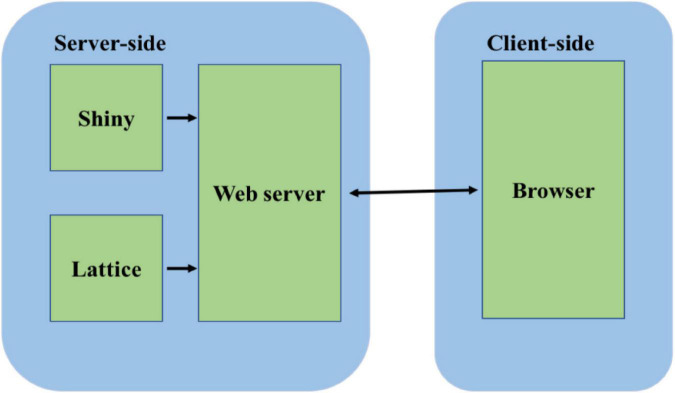
Software architecture of the simulator. The server side consists of the Shiny package and the Lattice package. The Shiny package is used for server-side computations. The Lattice package is used for graphical display. The computations and the graphs are served to the client browser through the webserver built into the Shiny package.

The Lattice package was used to create the graphs that were embedded into the webpage. The output of the web app is a set of voltage, current, and conductance graphs for the channels. These can be visualized instantaneously while changing the input values on the app after pressing the update button, or they can be downloaded as CSV tables and analyzed in spreadsheet software. The code is open-sourced and deposited at http://www.github.com/anuj2054/panama. The front end of the software is coded in a file *ui.R*, and the backend is coded in a file server as shown in Figure 1.

The app is hosted on a Shiny server located at the high-performance computing center facilities at Oklahoma State University. The computations for the equations all occur on the server’s side, so that there is no load on the user’s computer.

## Results and Discussion

There are three ways to access Panama. The first and the easiest way to access it is at http://www.neuronsimulator.com/. A second way that does not require a constant internet connection to work with the software is using the command runUrl^6^ on the R terminal. The command downloads the required files into an R folder and executes the software from the user’s local computer. A third way to access the app is using the command shiny::runGitHub (“panama,” “anuj2054”) on the R terminal given that the user has preinstalled Shiny. In the second and third methods, once the required command is run and the code is automatically downloaded to the local computer, access to the internet is not required anymore.

**FIGURE 2:**
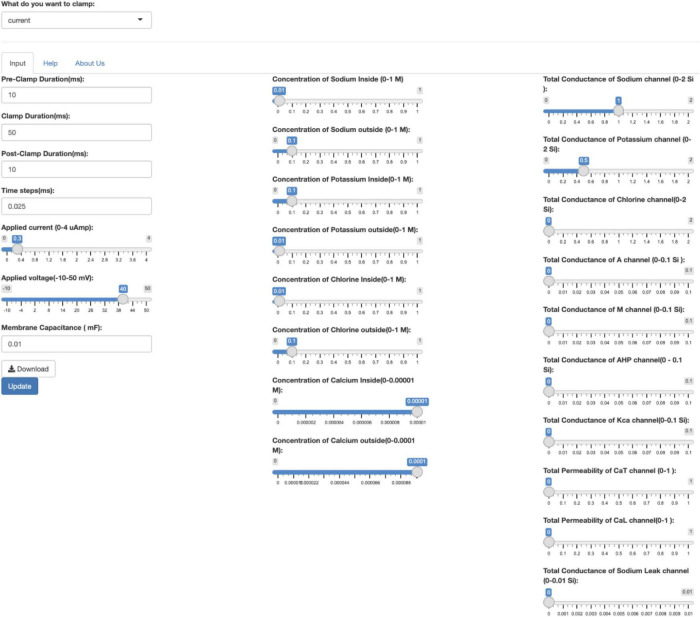
The input controls of the app on a desktop. The user first must select the current clamp or voltage clamp conditions from the drop-down menu. After selecting the clamping conditions, various parameters can be changed using the scrollbar. Pressing the Update button will update the output graphs depending on the values input. The user must click on Update each time they change the parameters. Clicking on the Download button will download the time series voltage, current and conductance of the ion channels in a CSV format.

On a desktop web browser, the input controls appear as in Figure 2.

However, on a mobile device, the three columns of the input controls are merged into one column for easy scrolling. On a mobile device, the use of sliders eases the process of entering values for individual parameters of the model. However, precision is not sacrificed. The user can change the input parameter values by hovering above the slider and using the keyboard to fine-tune the exact number they want to three significant decimal places. The default values on the app can be reloaded by refreshing the web browser.

**FIGURE 3:**
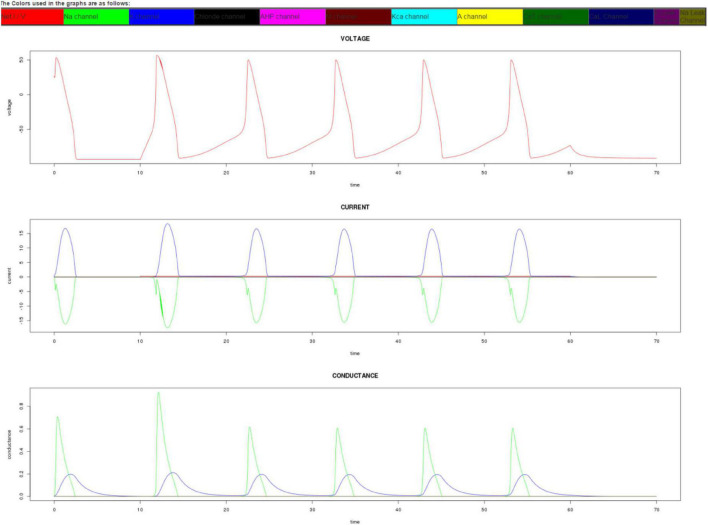
Output graphs of the app on a desktop. The simulator gives as output the time series representation of voltage, current, and conductance. The lines in the graph are color coded to represent different ion channels. In this graph, only sodium and potassium channels have been activated. The output graphs can also be replicated by downloading the CSV file and using a spreadsheet program to draw the graphs.

The app outputs conductance, voltage, and current data as both a graphical display and as downloadable CSV tables. The ability to download the output data as CSV tables enables the user to use their own spreadsheet software, such as Excel, to further analyze the data or embed the graphs in their own documents. On a desktop web browser, the output graphs appear as shown in Figure 3.

Each of the lines inside the graphs is color-coded and described with the name of the channel inside its respective colored rectangle. During the current-clamp mode, the current injection can be made more noticeable in the current graph by increasing the applied current and observing the steep red line that appears after the preclamp duration. The user can increase the clamp duration to see numerous action potentials that resemble neural spikes.

When using the app for educational purposes, it is encouraged to use it in conjunction with a textbook about ion channels and their electrical properties. The default ion concentrations and channel conductance can be changed to those for different types of cells, such as squid axon cells, and observe its effect on the current and voltage of the cell. The default values used in the app are for a mammalian cell at room temperature (Lodish et al., 2008). This app has been particularly helpful in pointing out the reasons for the changes observed in the action potentials when different types of channels are activated. Changing the default capacitance and conductance values demonstrates how different conditions of a cell membrane affect the electrical properties of the cell. This practice gives a hands-on approach toward learning neurophysiology that would otherwise only get from a textbook or from an expensive electrophysiology rig.

The numerical output of the simulator was tested against NEURON with similar parameters. Both programs returned equivalent results. The app was also tested under different operating systems (Windows, Android, iOS, Mac, and Linux) and under different browsers (Chrome, Firefox, and Internet Explorer). It was found to operate consistently across all platforms. This app can be used by educators, students of pharmacology, physiological science, neurobiology, and neuroscience, who are interested in simulating particular ion channels and in knowing their physiological properties so that it can be used to understand the physiological properties of voltage-gated ion channel, which acts as a triggering signal for various pathological conditions.

Future versions of the app will have phase space graphs to help users better understand membrane dynamics. It will also model synaptic currents where the chloride channels would play an important role. A better help section, tutorials, and an even cleaner user interface is also being planned.

Panama is a first-of-its-kind, a touch-friendly, mobile-friendly online tool that models electrophysiology of 11 different types of ion channels using Hodgkin–Huxley-style differential equations. It requires no user training for installation and no infrastructure for downloading, which makes it suitable for educational purposes. Virtual learning has been an important pedagogical tool in the physical and biological sciences. Even though the science of physics and chemistry has benefitted from having a wealth of virtual simulation tools for education, biology still lags behind in the use of such tools for education. Such portable apps can act as a virtual laboratory where there is a lack of physical resources in classrooms to purchase a high-cost electrophysiology workstation. Virtual simulation environments can improve the quality of education by providing computer-based skills in developed countries at a minimal cost. In future, such apps can provide beneficial web training by means of Massive Open Online Courses (MOOCs). Such tools should also be expanded for other areas of biological studies, such as cell biology, ecology, and population studies.

## Data Availability Statement

The datasets presented in this study can be found in online repositories. The names of the repository/repositories and accession number(s) can be found in the article/Supplementary Material.

## Author Contributions

AG and BR conceived, designed the software, performed the analysis, and wrote the manuscript. Both authors contributed to the article and approved the submitted version.

## Conflict of Interest

The authors declare that the research was conducted in the absence of any commercial or financial relationships that could be construed as a potential conflict of interest.

## Publisher’s Note

All claims expressed in this article are solely those of the authors and do not necessarily represent those of their affiliated organizations, or those of the publisher, the editors and the reviewers. Any product that may be evaluated in this article, or claim that may be made by its manufacturer, is not guaranteed or endorsed by the publisher.

## Footnotes

1
http://www.cs.cmu.edu/∼dst/HHsim/

2
http://vlab.amrita.edu/?sub=3&brch=43

3
http://sites.lafayette.edu/schettil/neurolab/

4
https://phet.colorado.edu/en/simulation/neuron

5
http://nerve.bsd.uchicago.edu/

6
https://github.com/anuj2054/panama
