# Supplementary material for: Panama: An Open-Source Educational App for Ion Channel Biophysics Simulation
Source: Front Neuroinform. 2022 Mar 9;16:813940. doi: 10.3389/fninf.2022.813940 (PMC8959878; doi:10.3389/fninf.2022.813940)
Supplement: Supplementary file 1 [file Data_Sheet_1.docx]

**Panama: An open-source educational app for ion channel biophysics simulation**

Binita **Rajbanshi^1^,** Anuj **Guruacharya^2^**

^1^Department of Epileptology, University Hospital Bonn, Bonn, Germany

^2^Department of Biology, University of Oklahoma, Norman, USA

**Corresponding Author:** Anuj Guruacharya, PhD (email: [anuj2054@gmail.com](mailto:anuj2054@gmail.com))

###### **Supplementary equations**

Equations 3 to 13 described the individual ionic currents for each channel used in the simulator. The electrochemical gradients driving the flow of ions were represented as voltage sources (*E_n_*) whose voltages were determined by Nernst equations that are dependent on the ratio of extra- to intra-cellular concentrations of the ionic species of interest. All gating variables, *m*, *n*, *h, m_KCa_, m1_A_ , m2_A_, h1_A_, h2_A_, m_AHP_, m_M_, m_T_, m*_L_ were dimensionless numbers, ranging from 0 to 1. *m* denotes the probability of finding a channel in its open/permissive state and 1-*m* denotes the probability of it being closed or in a non-permissive state (current flow will be zero in this state). Other gating variables followed a similar representation. *InCa* and *OutCa* represented the concentration of calcium ions inside the cell and outside the cell in mM respectively. The constant values used in Equations 5 to 16 which cannot be changed by the user are the temperature (*T*) set to be a room temperature of 293.15 Kelvins, gas constant (R) set to 8314 Joules/mole/kelvin, and the Faraday constant (*F*) set to 96485 Ampere-seconds/mole.

_
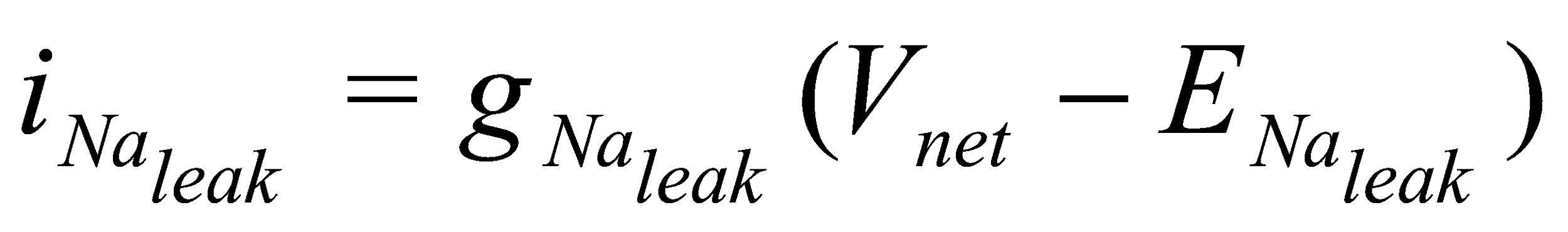
_ (3)

_
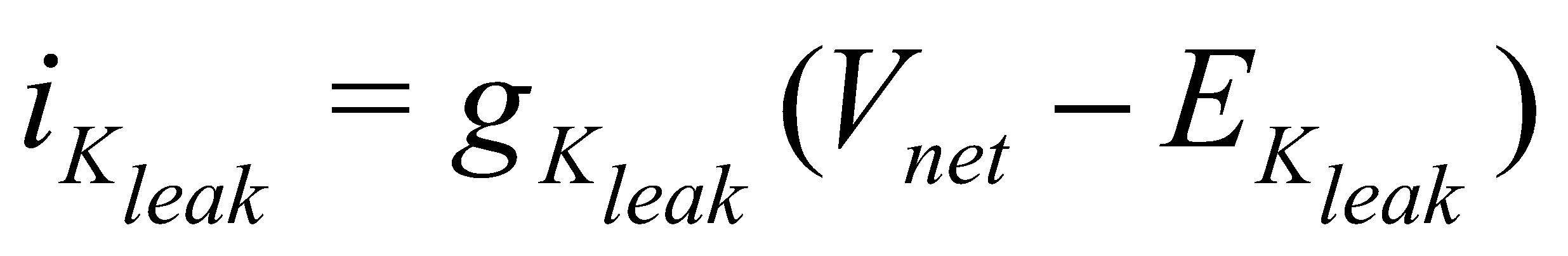
_ (4)

_
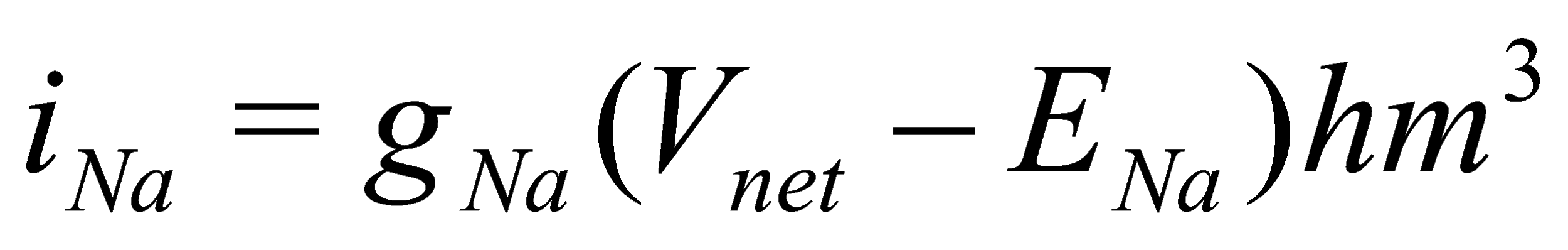
_ (5)

_
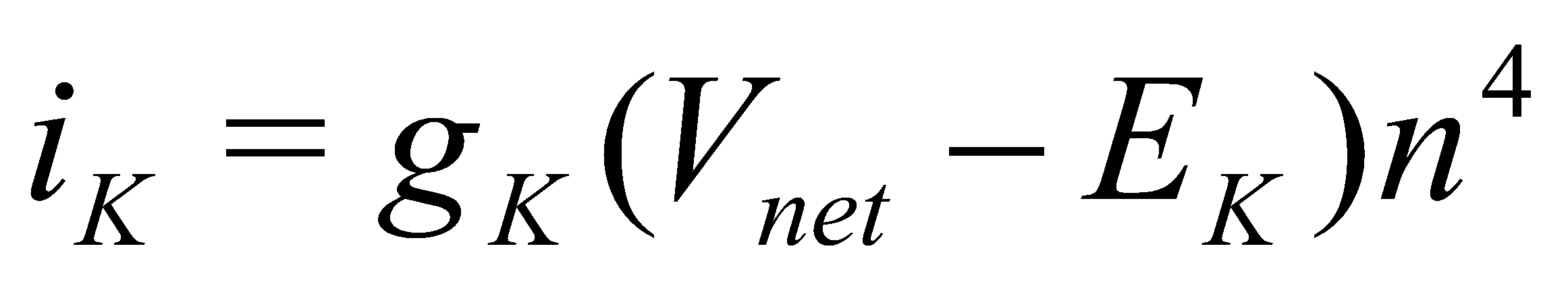
_ (6)

_
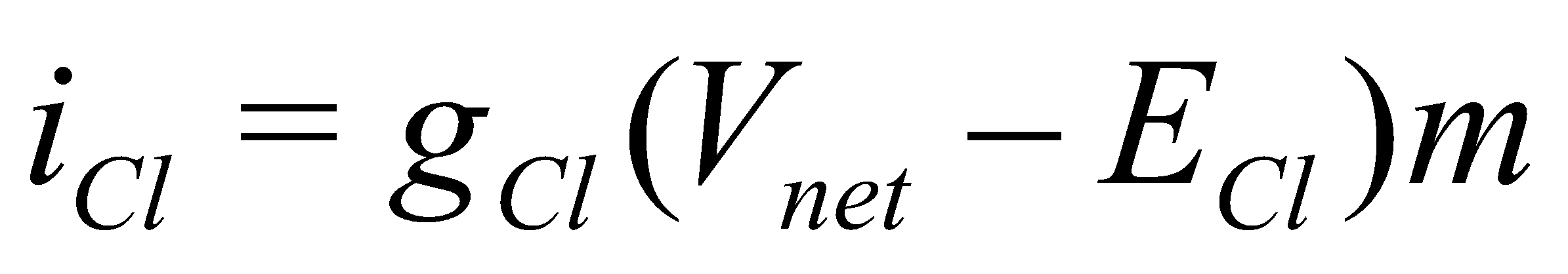
_ (7)

_
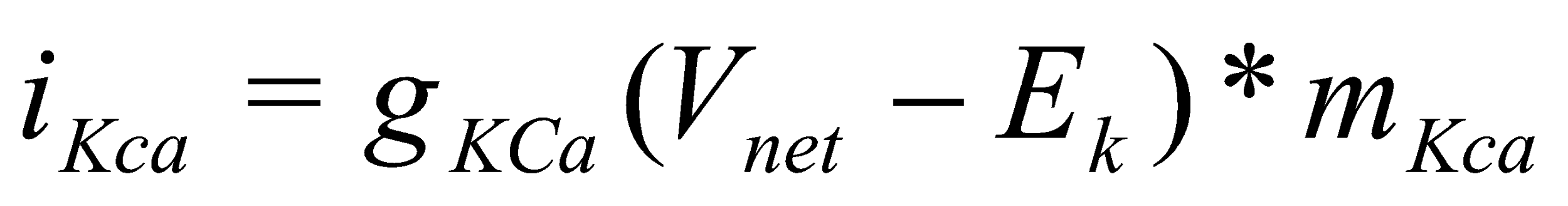
_ (8)

_
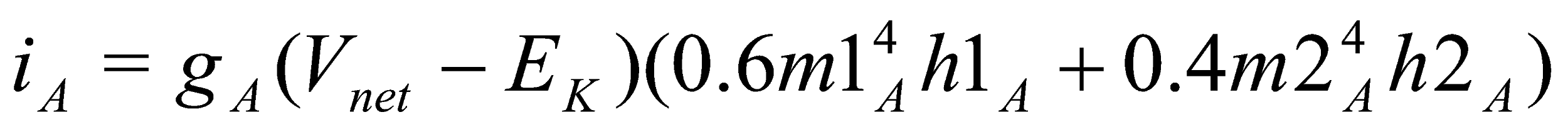
_ (9)

_
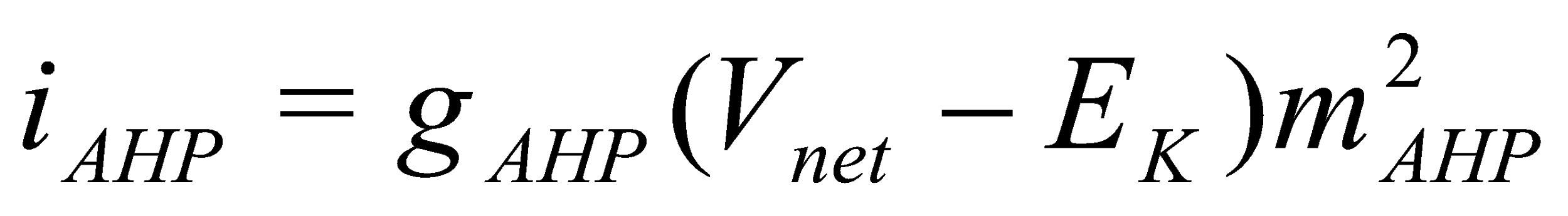
_ (10)

_
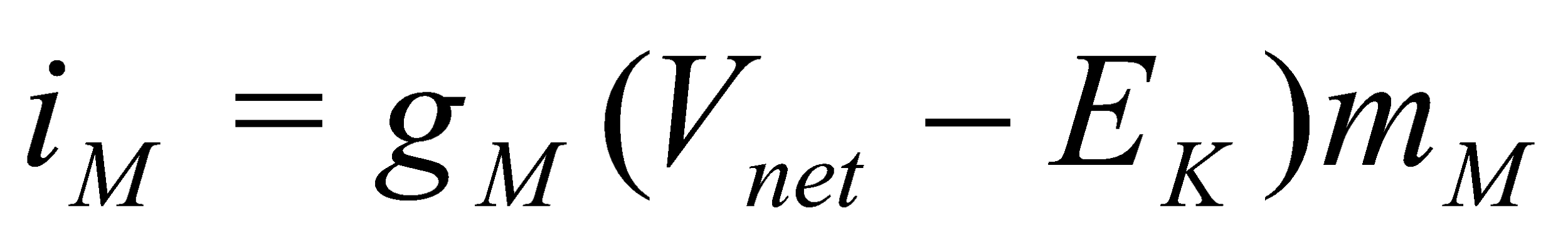
_ (11)

_
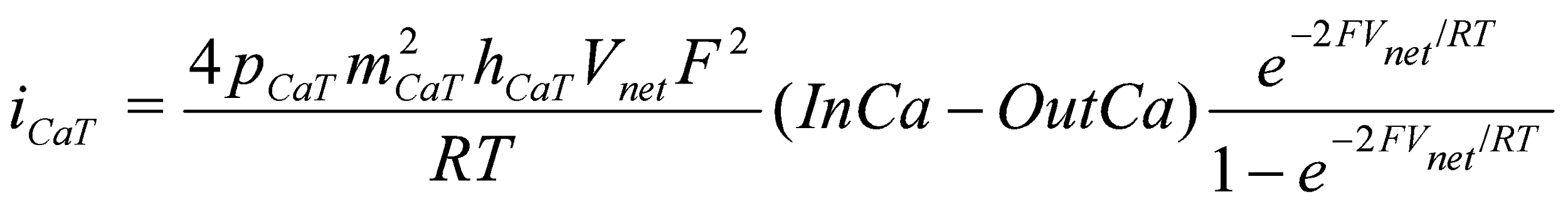
_ (12)

_
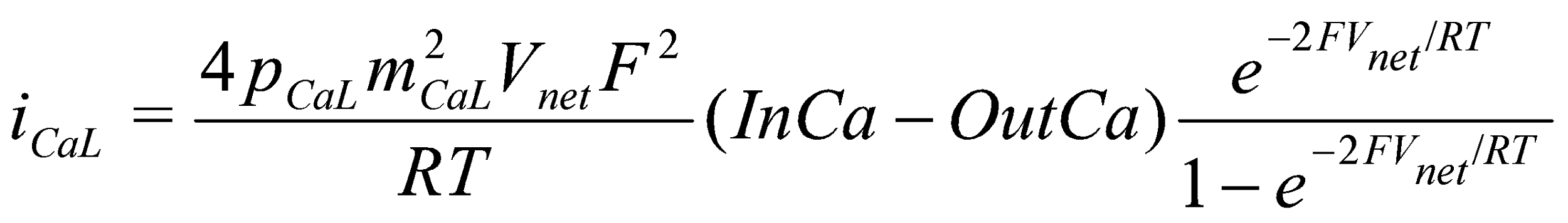
_ (13)

Differential equations in Equations 14 to 27 were used to model the activation and deactivation of the gating variables. The differential equations were discretized using an explicit forward Euler algorithm with a default time step of 0.025 ms. The time step can be changed by the user; however, it is not recommended to turn it lower than the default value since it can slow down the simulation. The equations were assumed to be initial value problems and solved for each value of voltage across the membrane. So, these differential equations were solved with different values of *V_net_* across the membrane.

_
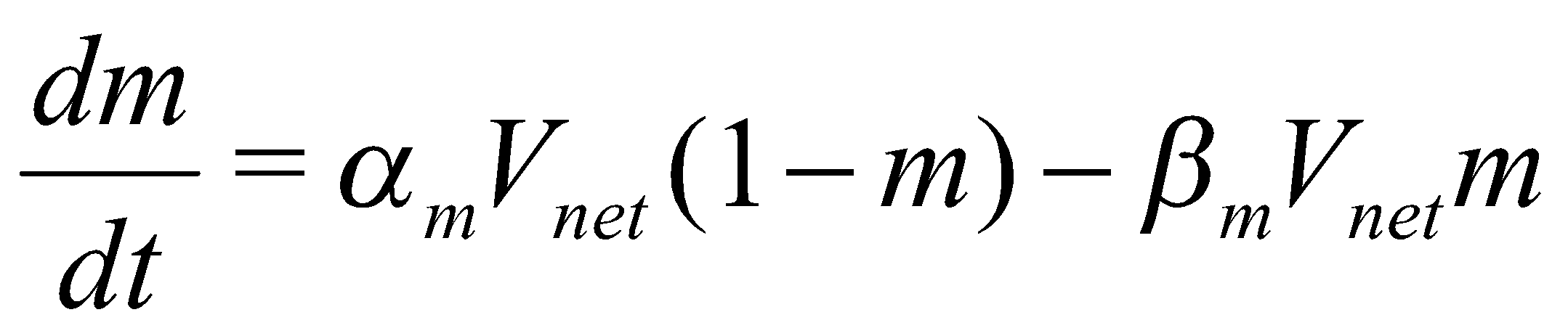
_ (14)

_
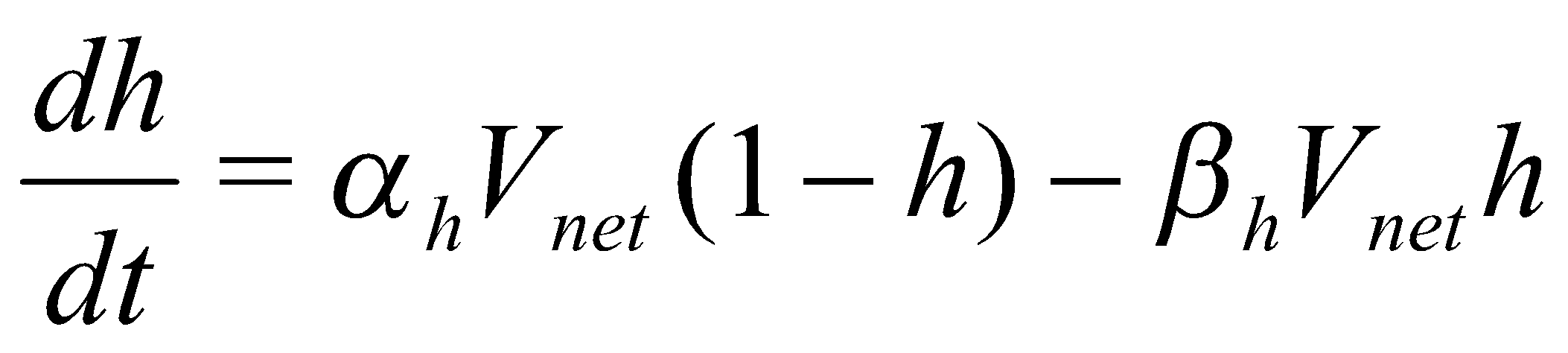
_ (15)

_
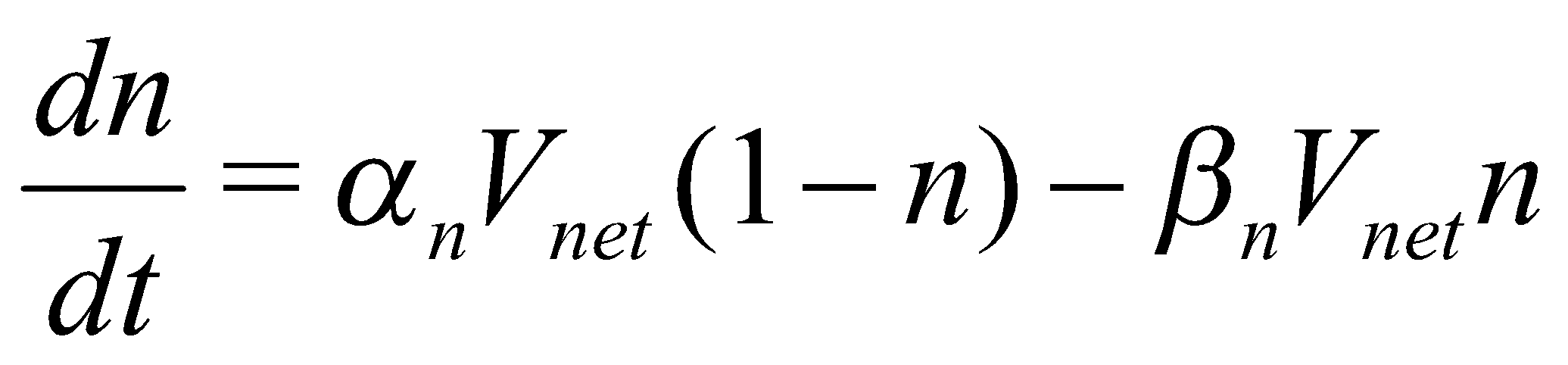
_ (16)

_
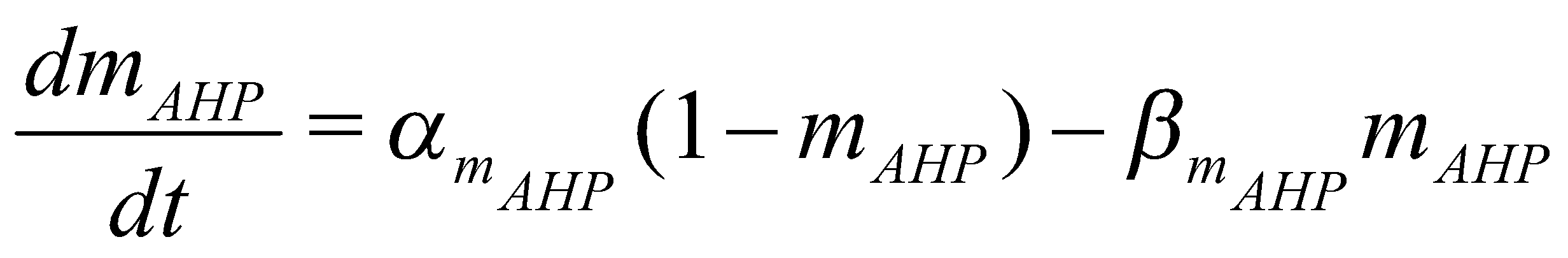
_ (17)

_
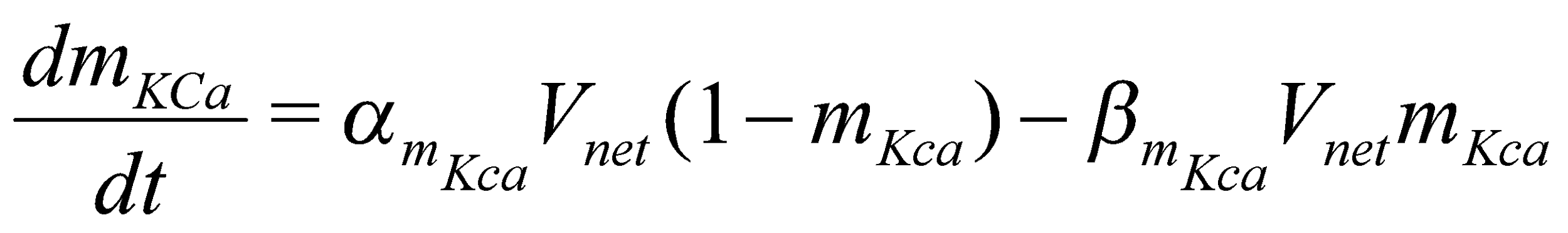
_ (18)

_
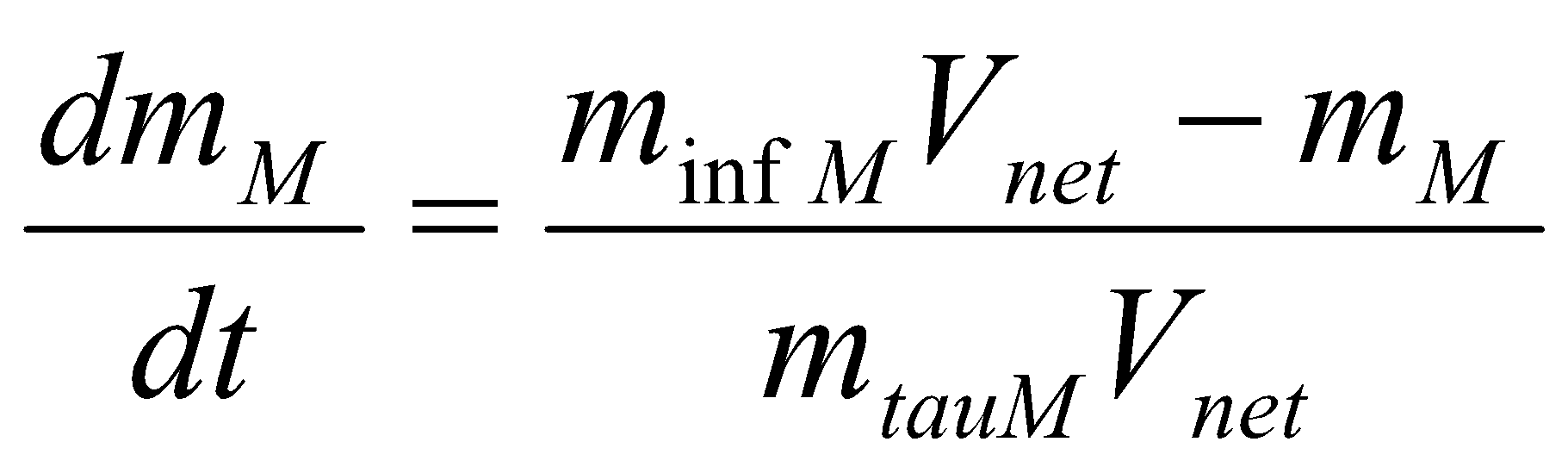
_ (19)

_
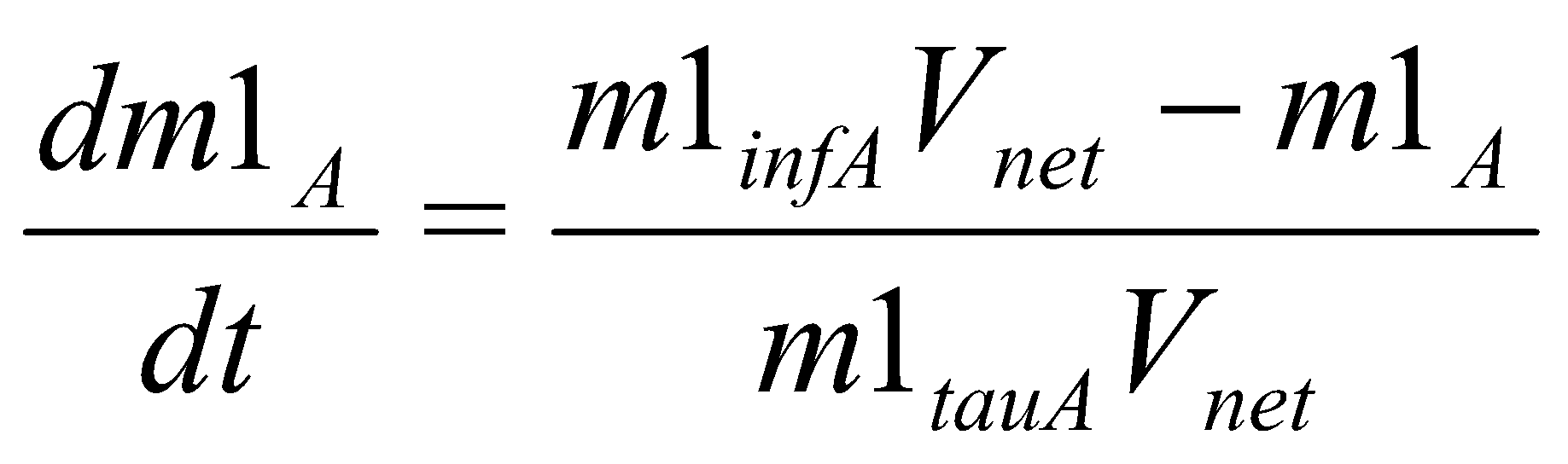
_ (20)

_
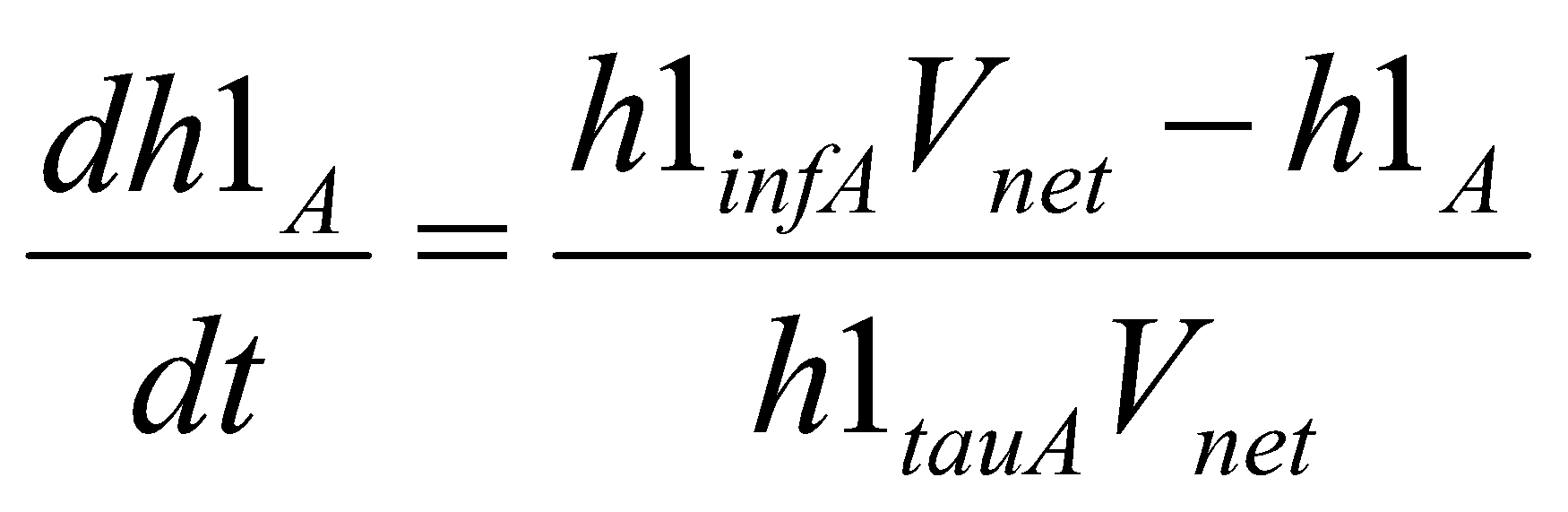
_ (21)

_
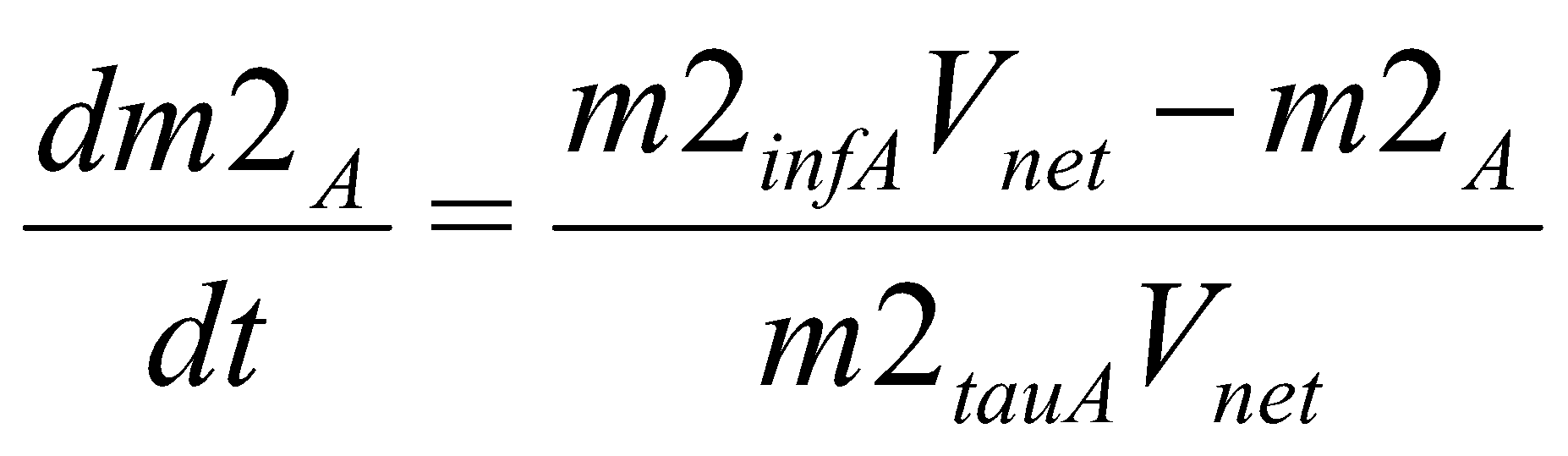
_ (22)

_
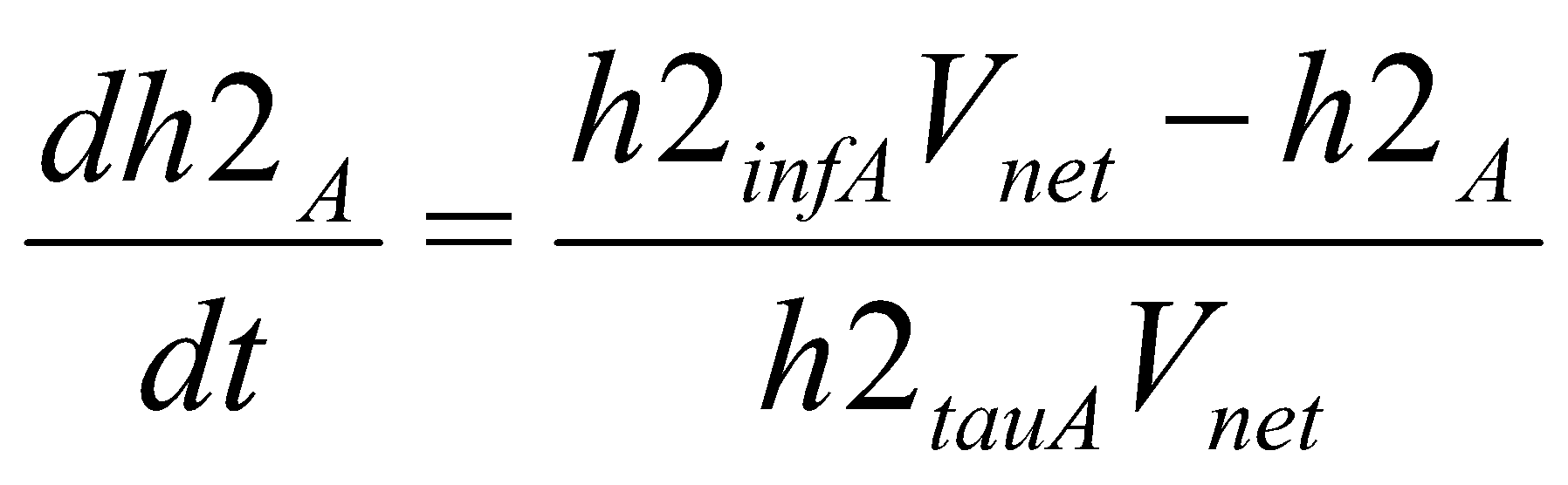
_ (23)

_
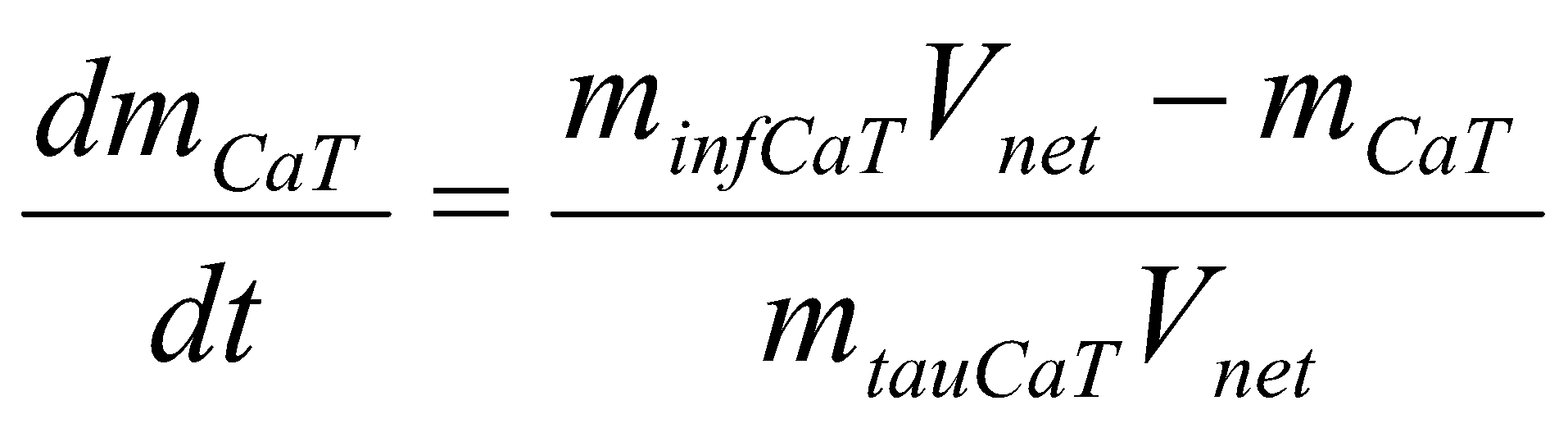
_ (24)

_
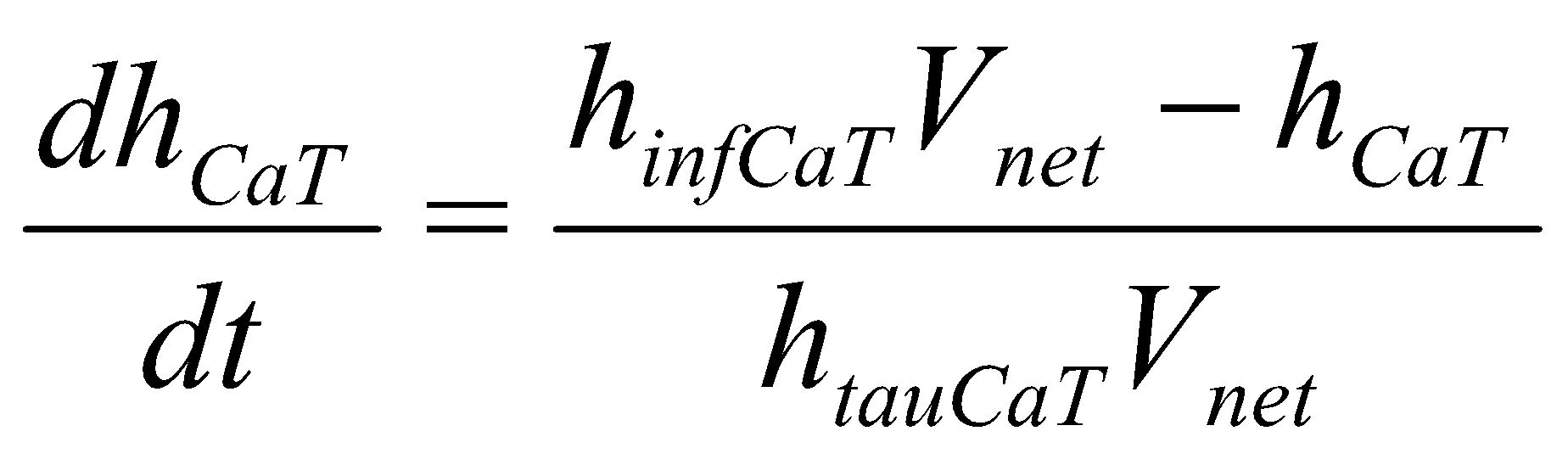
_ (25)

_
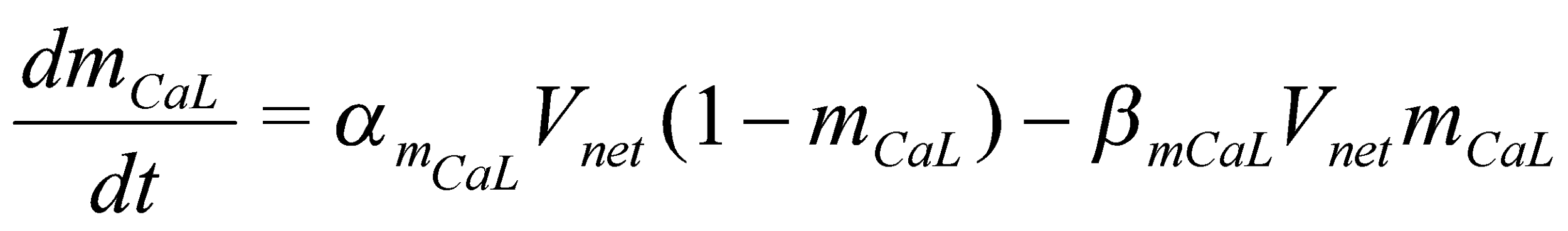
_ (26)

Equations 27 to 54 were used to model the rate constants used and the steady state values of the gating variables in Equations 14 to 26. These equations are dependent on the membrane voltage. For the initial value of *V_net_* and *I_net_*, default values of 40 mVolt and 0.3 nAmp were used.

_
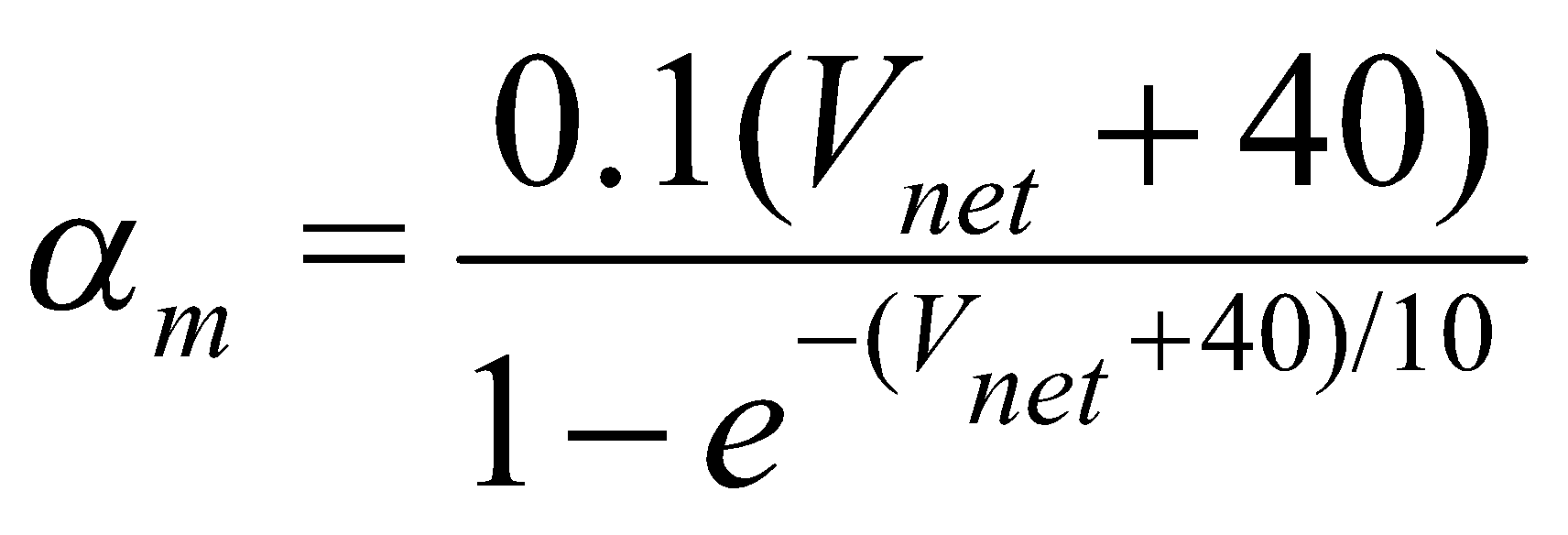
_ (27)

_
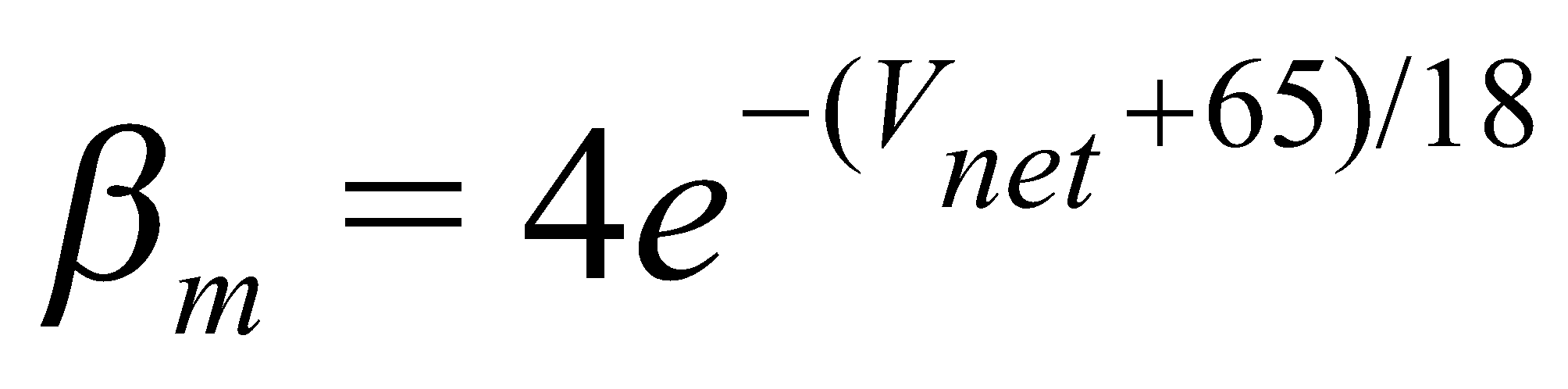
_ (28)

_
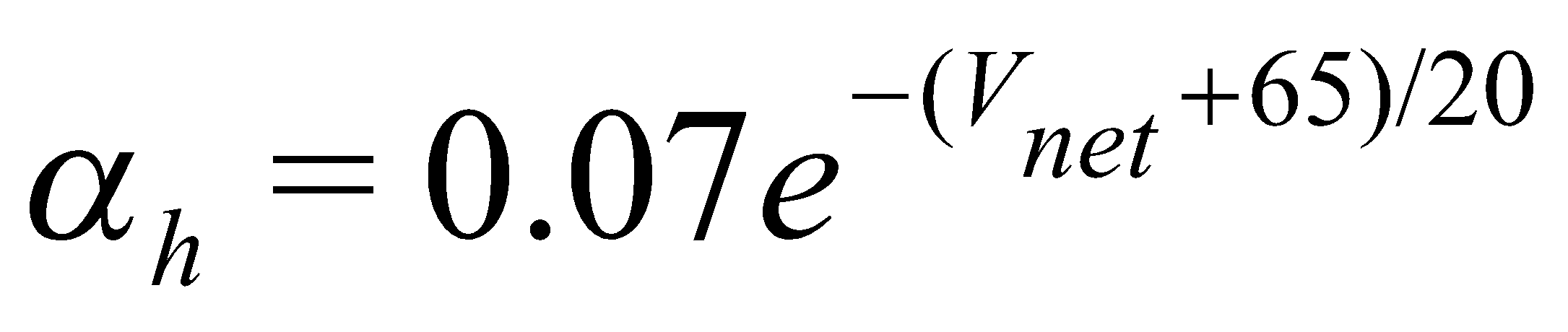
_ (29)

_
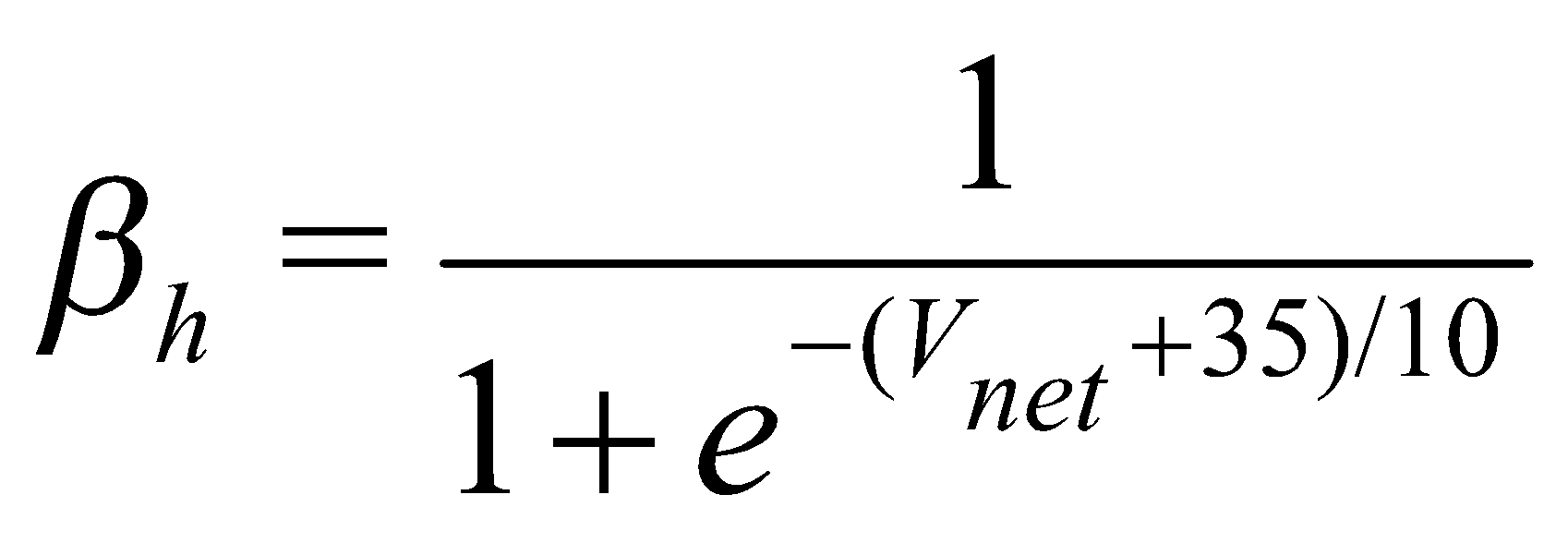
_ (30)

_
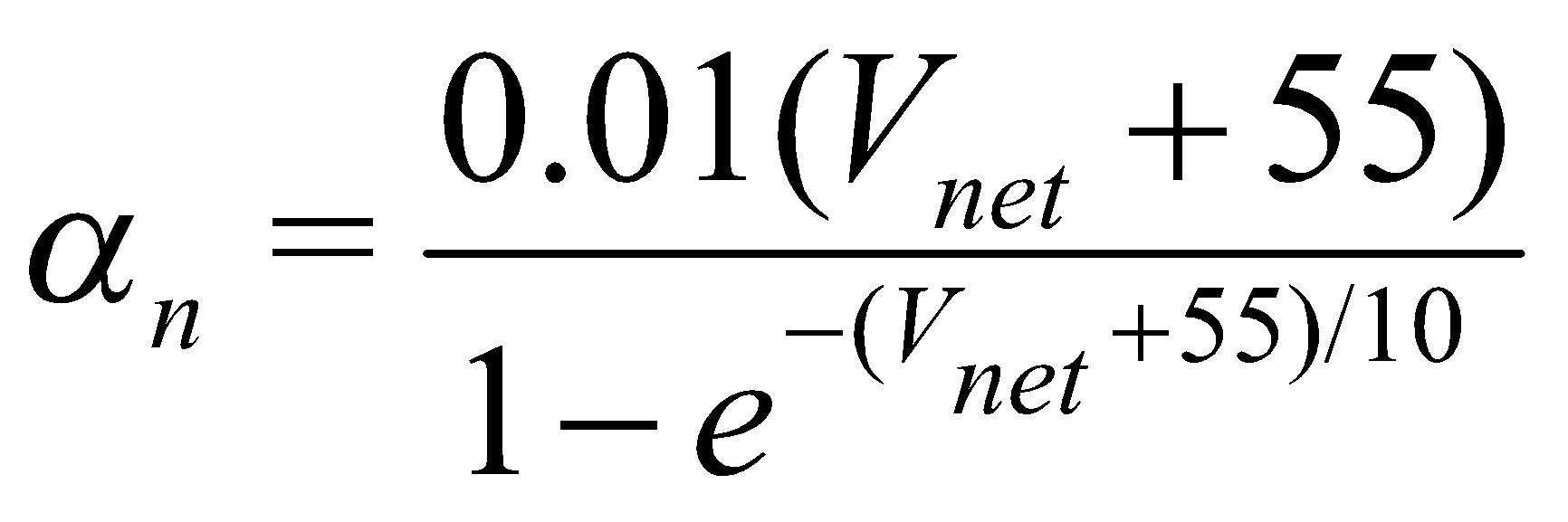
_ (31)

_
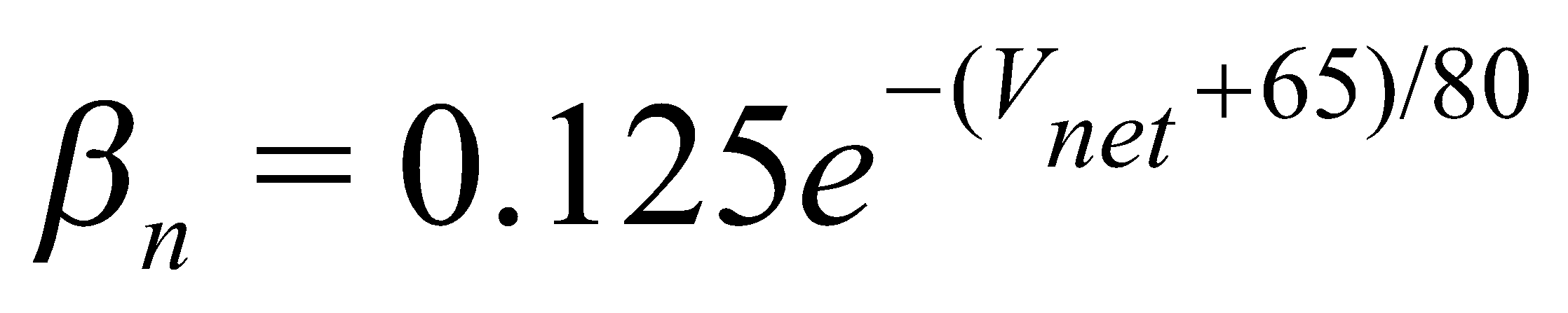
_ (32)

_
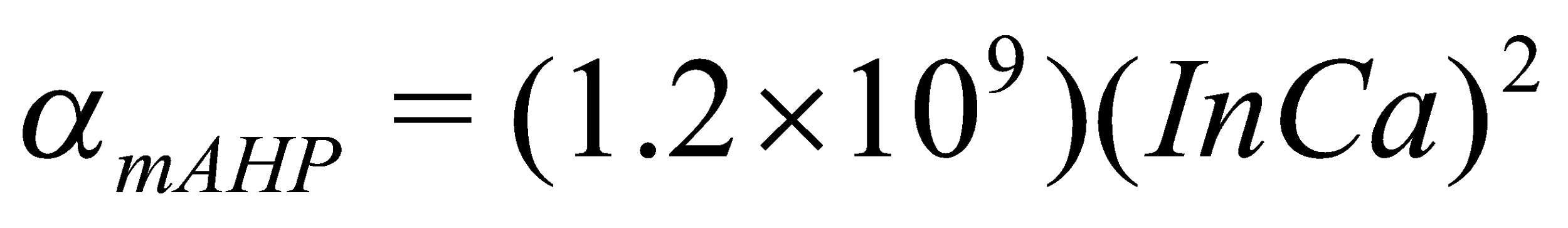
_ (33)

_
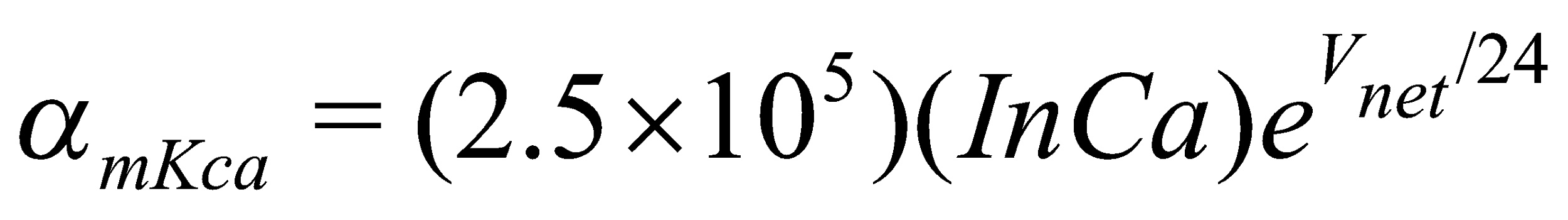
_ (34)

_
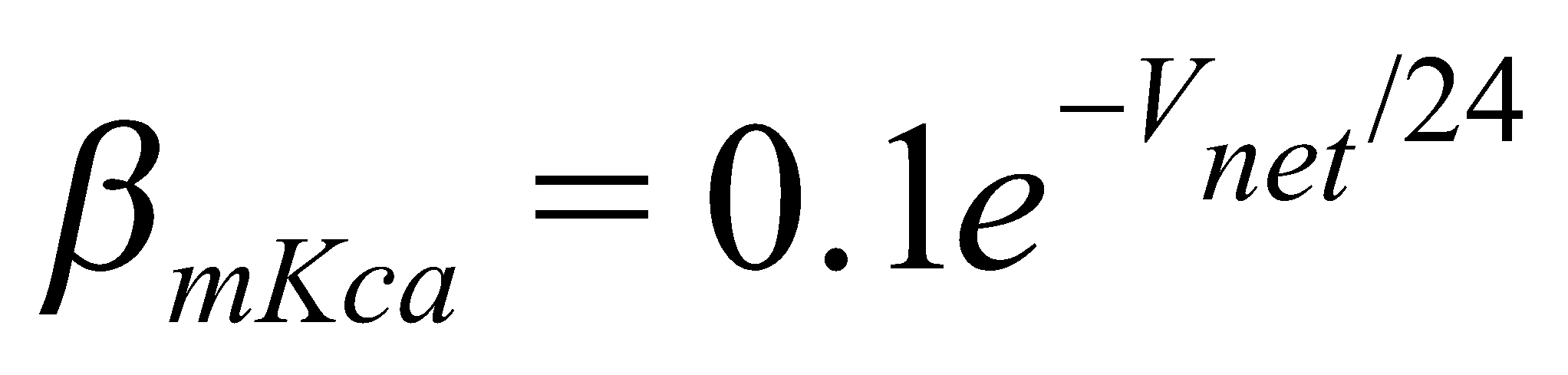
_ (35)

_
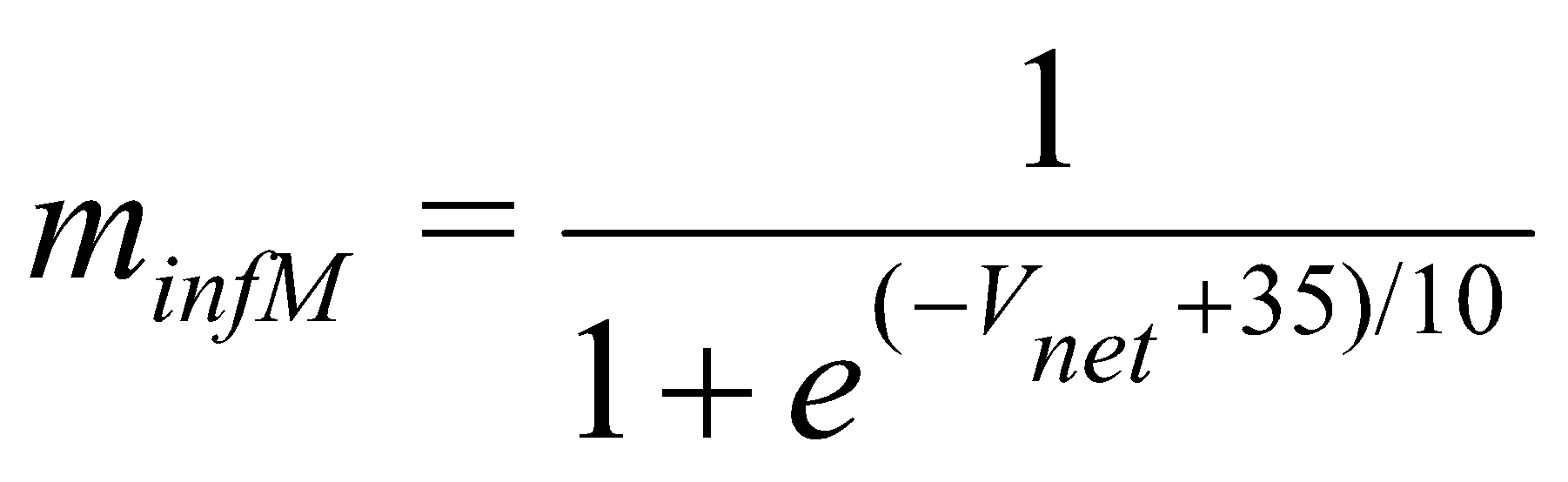
_ (36)

_
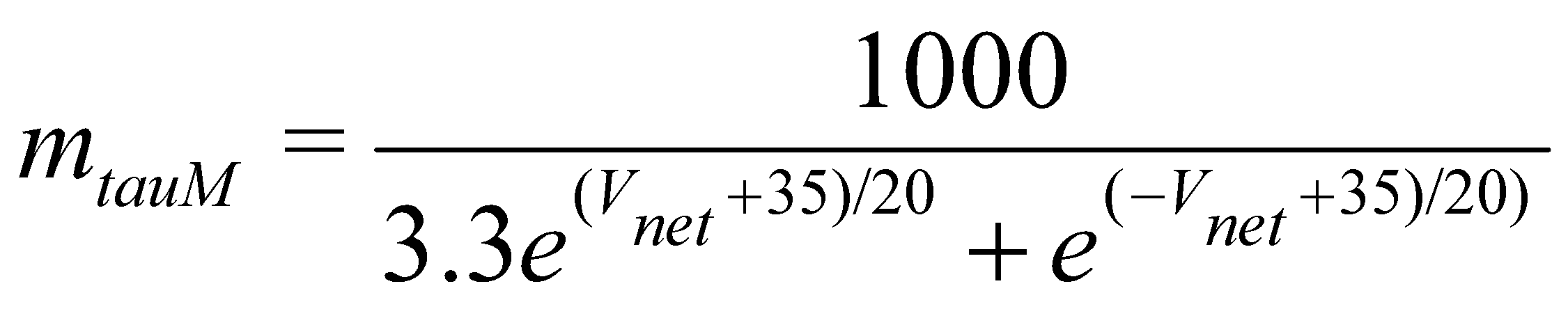
_ (37)

_
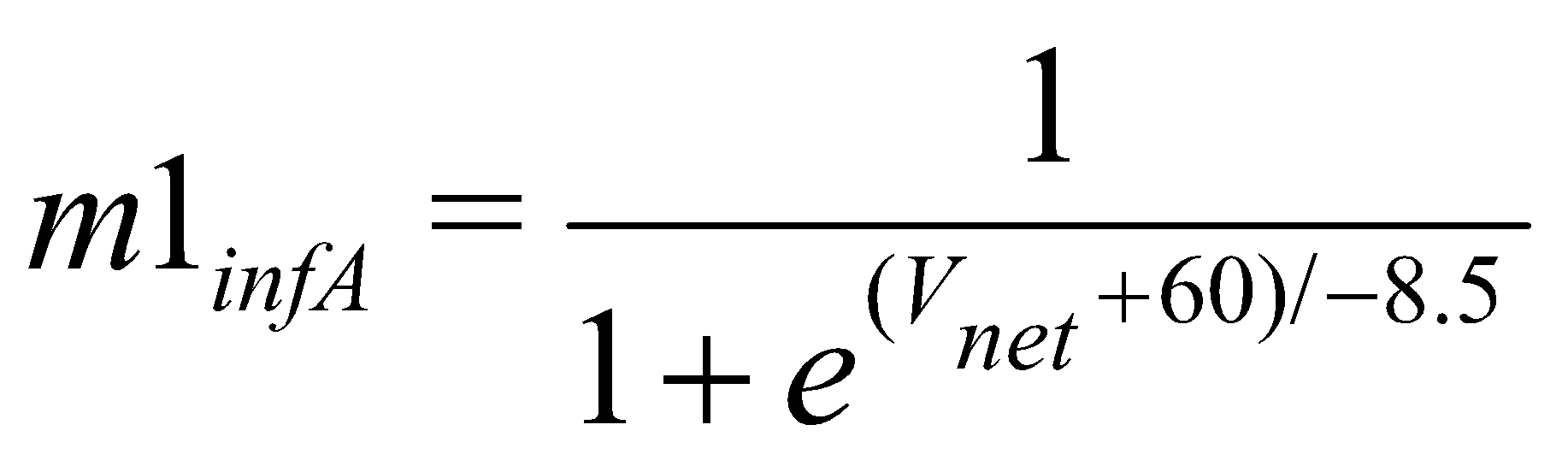
_ (38)

_
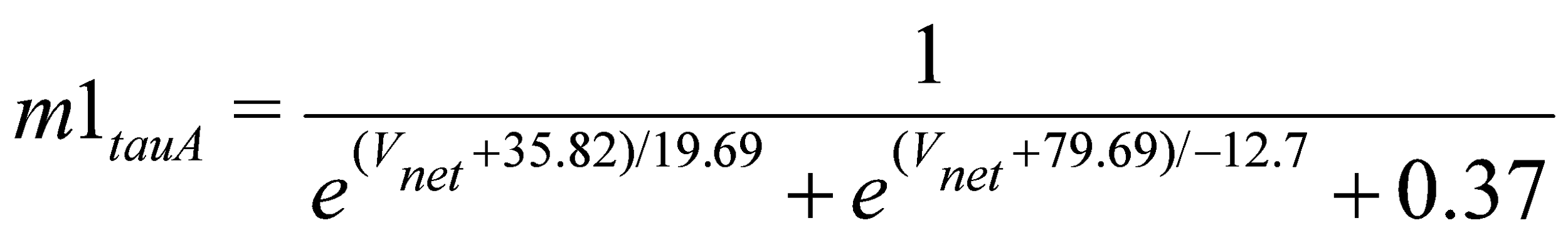
_ (39)

_
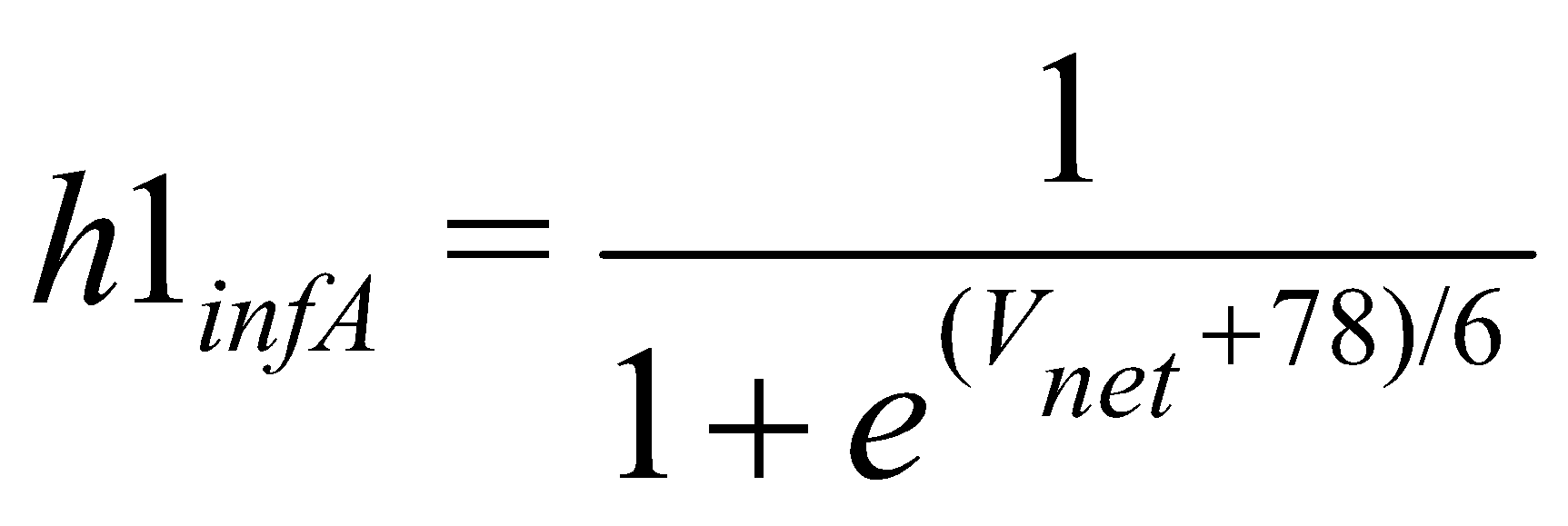
_ (40)

_
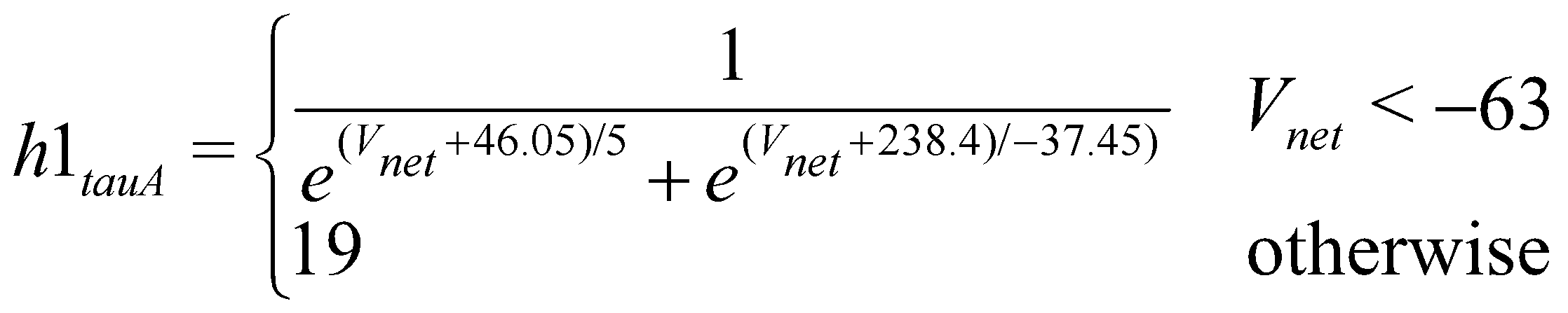
_ (41)

_
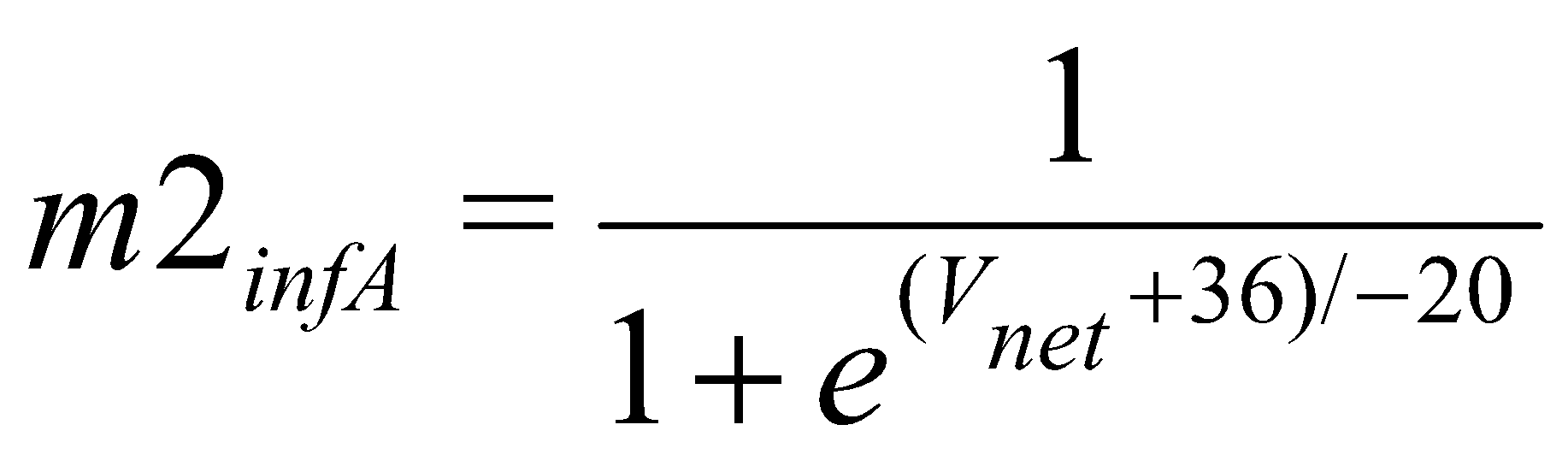
_ (42)

_
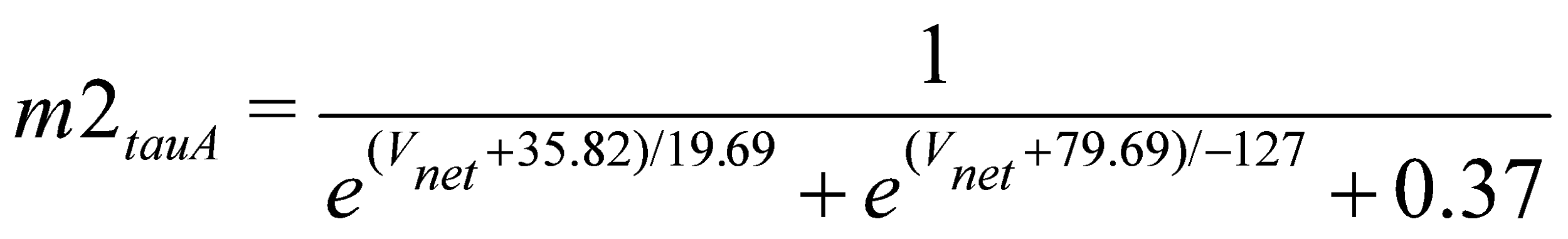
_ (43)

_
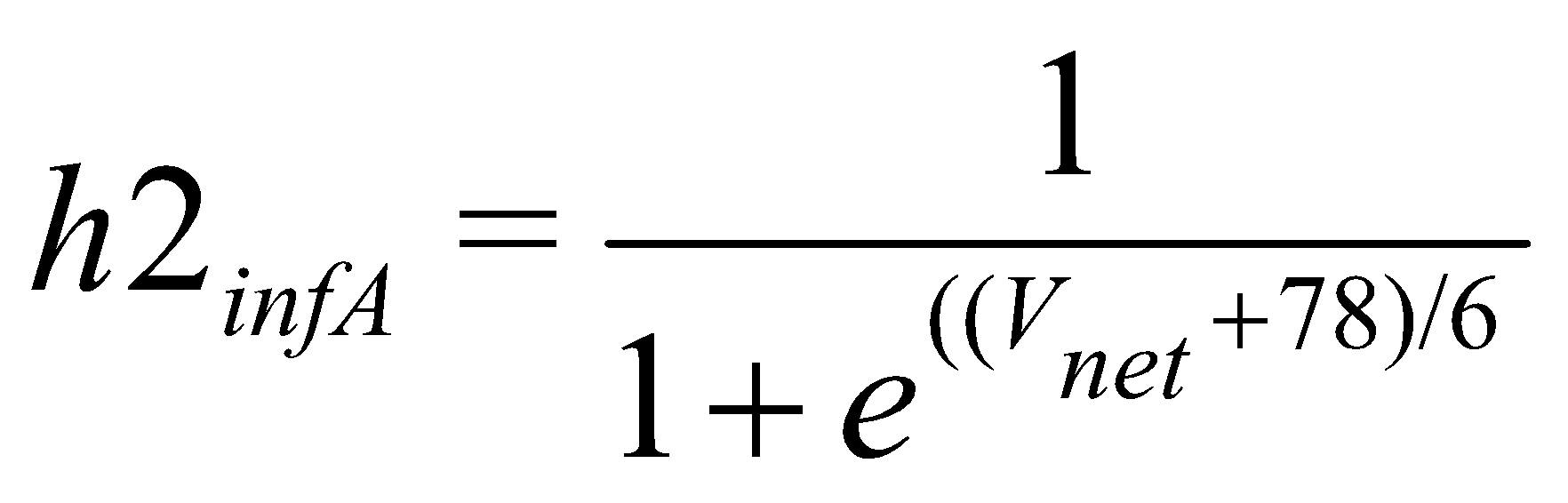
_ (44)

_
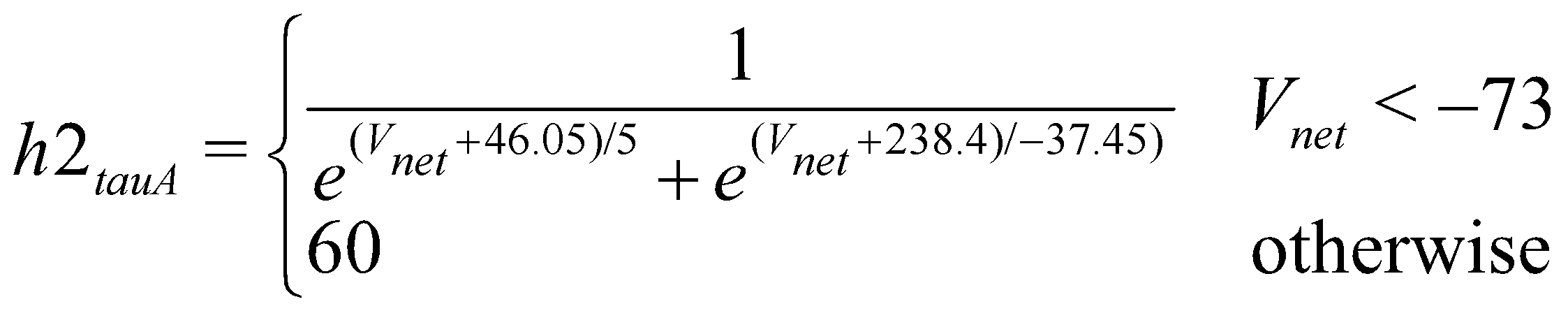
_ (45)

_
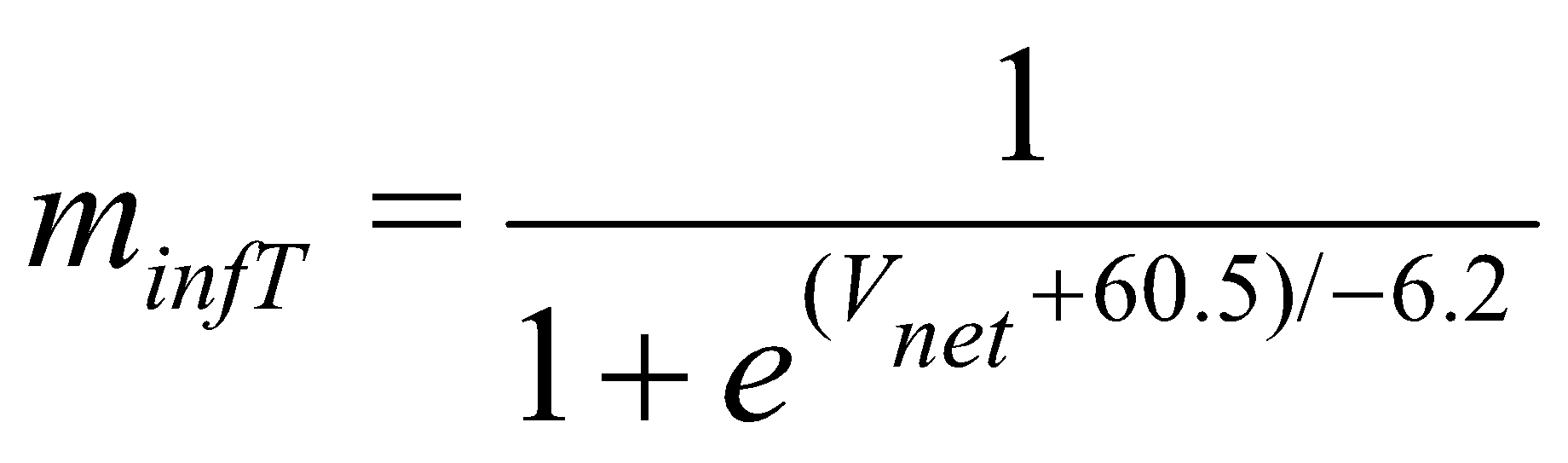
_ (46)

_
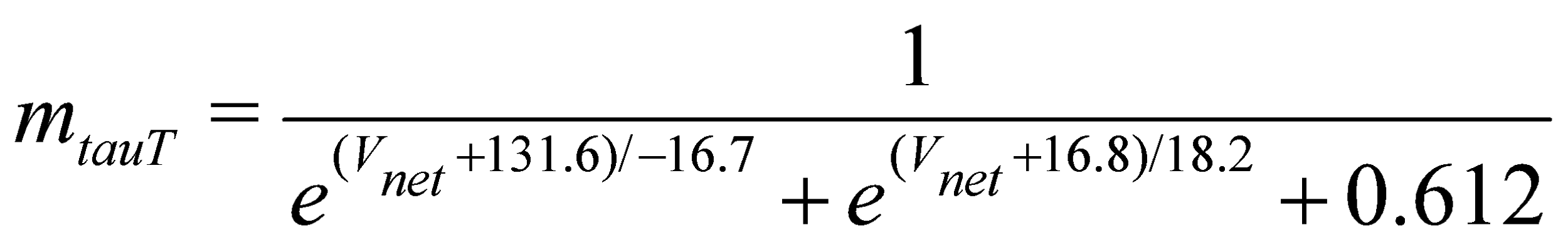
_ (47)

_
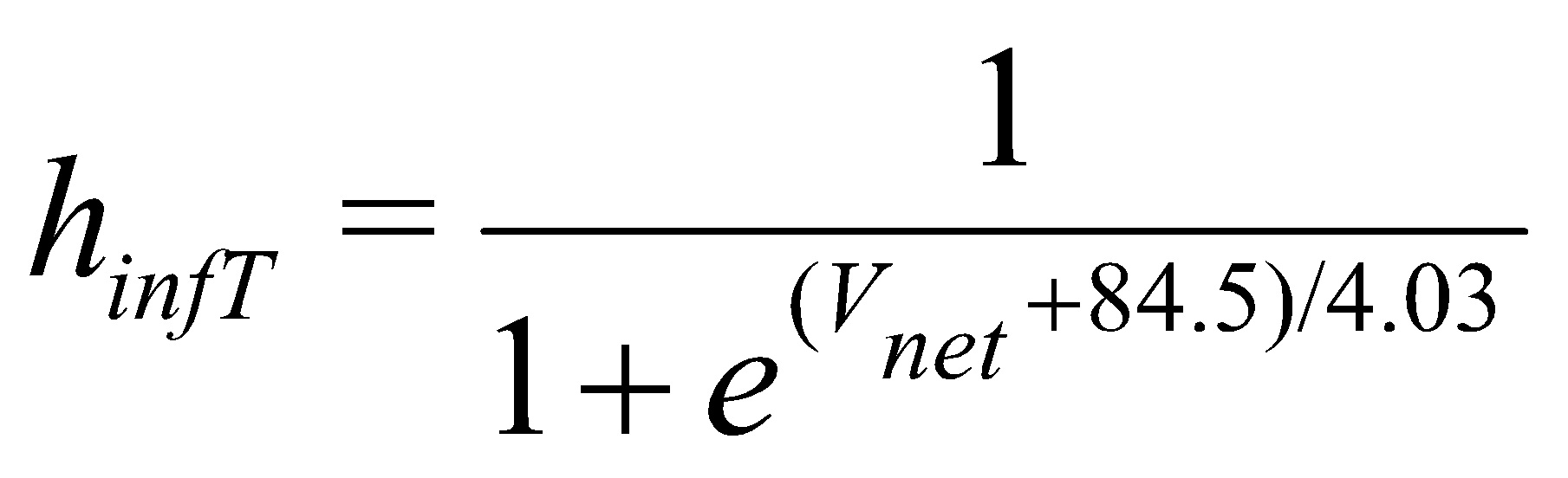
_ (48)

_
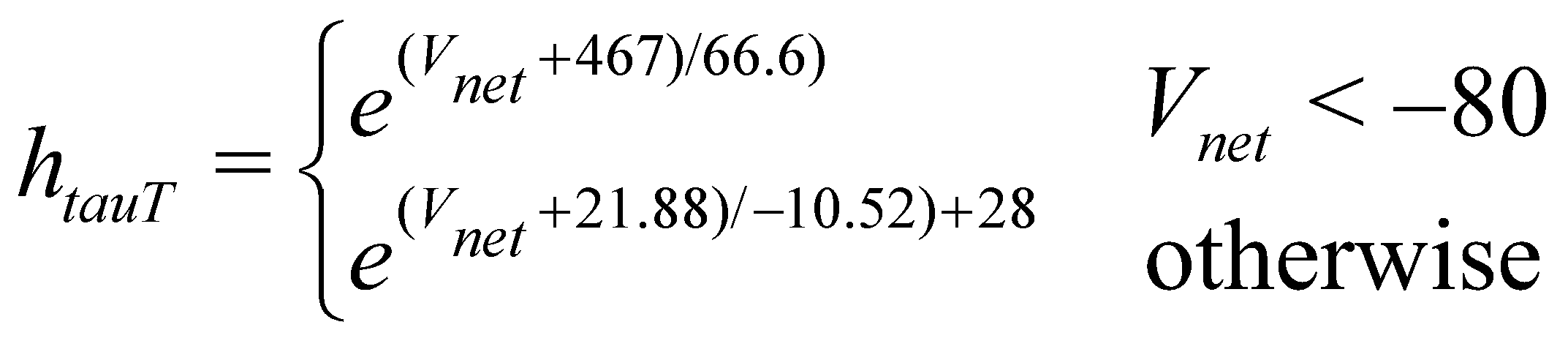
_ (49)

_
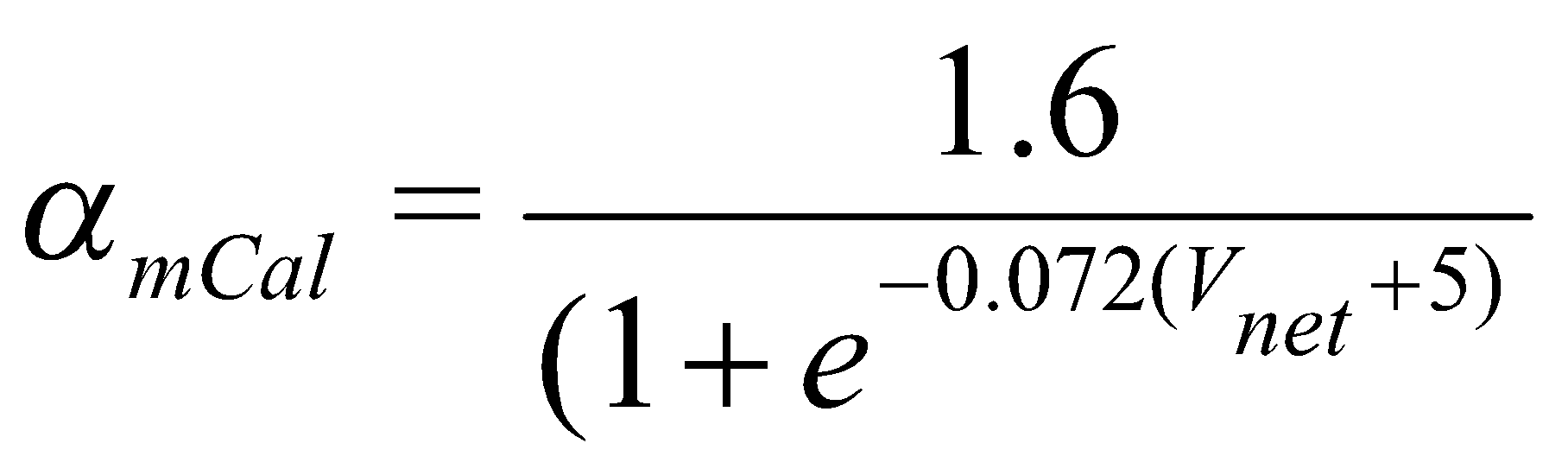
_ (50)

_
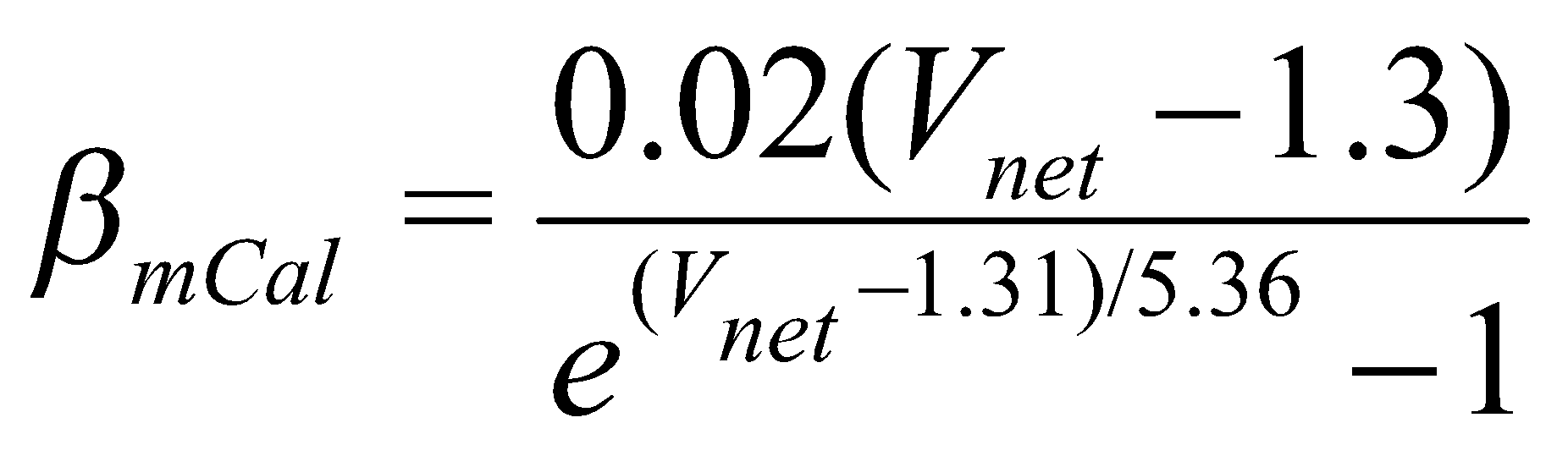
_ (51)

_
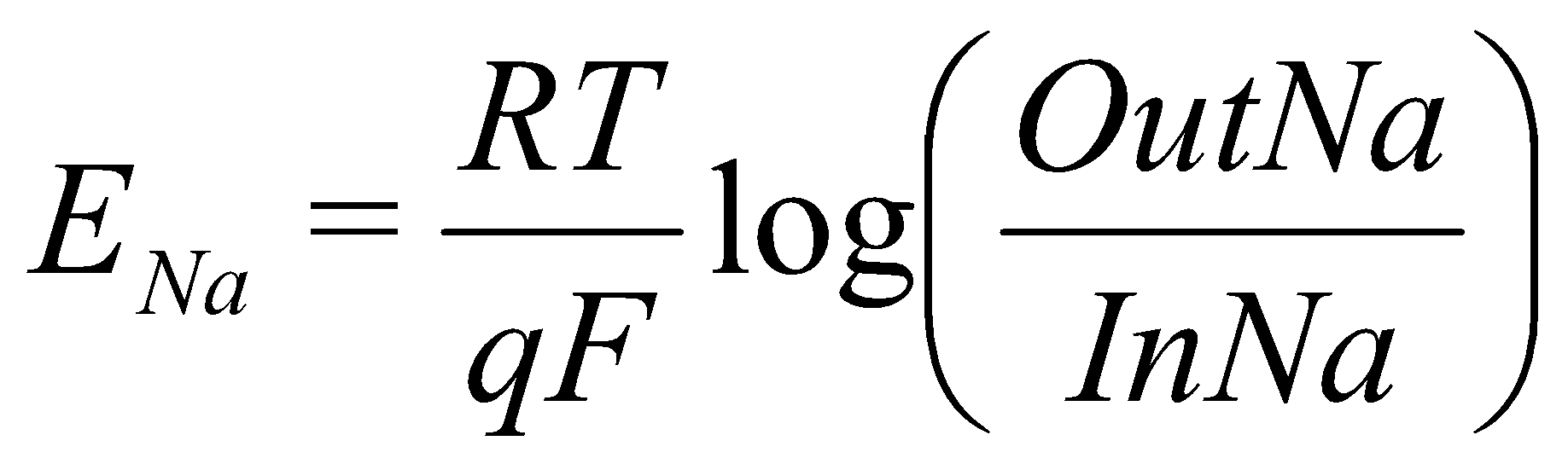
_ (52)

_
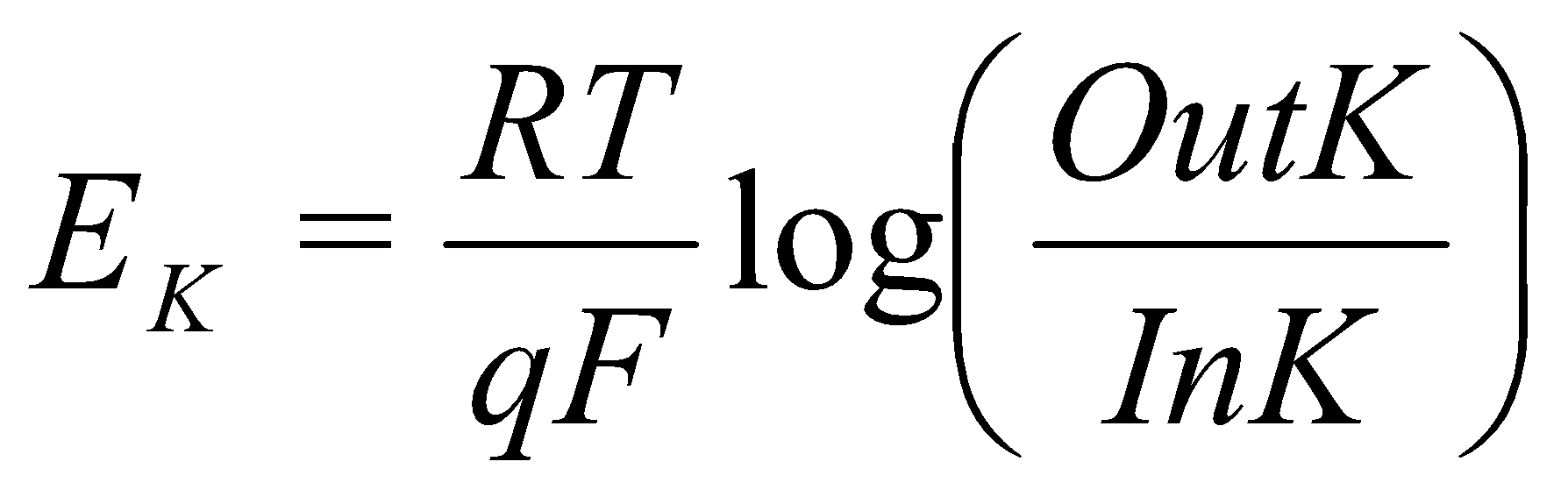
_ (53)

_
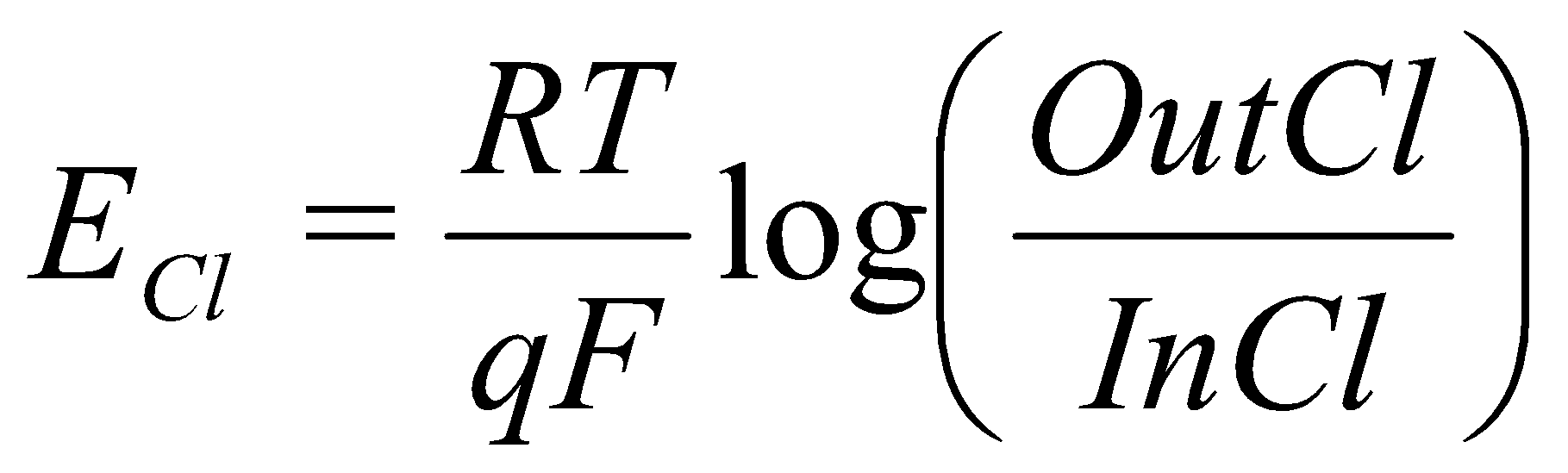
_ (54)
